# Cancer-associated fibroblast-derived extracellular vesicles promote lymph node metastases in oral cavity squamous cell carcinoma by encapsulating ITGB1 and BMI1

**DOI:** 10.1186/s12885-024-11855-0

**Published:** 2024-01-22

**Authors:** Tianzhu Lv, Hongjing Liu, Ling Mao, Yanrong Song, Lili Liao, Kun Zhong, Binbin Shuai, Yingkun Luo, Tingting Guo, Wentao Huang, Shenyingjie Zhang

**Affiliations:** 1 Guizhou Medical University, 550004 Guiyang, Guizhou P.R. China; 2 China-British Joint Molecular Head and Neck Cancer Research Laboratory, Stomatological Hospital of Guizhou Medical University, 550004 Guiyang, Guizhou P.R. China; 3 Comprehensive Emergency Department of Stomatology, Stomatological Hospital of Guizhou Medical University, 550004 Guiyang, Guizhou P.R. China; 4 School of Savaid Stomatology, Hangzhou Medical College, 311399 Hangzhou, Zhejiang P.R. China; 5 Medical Department, The First Affiliated Hospital of Zhejiang Chinese Medical University (Zhejiang Provincial Hospital of Chinese Medicine), 310006 Hangzhou, Zhejiang P.R. China

**Keywords:** Oral cavity squamous cell carcinoma, Lymph node metastasis, Cancer-associated fibroblast-derived extracellular vesicles, BMI1, ITGB1

## Abstract

**Background:**

Extracellular vesicles (EVs) have been revealed to facilitate the development of oral squamous cavity cell carcinoma (OCSCC), while its supporting role in lymph node metastases is under continuous investigation. This study aimed to examine the function of cancer-associated fibroblasts (CAF)-derived EVs (CAF-EVs) during lymph node metastasis in OCSCC and the mechanisms.

**Methods:**

CAF were isolated from OCSCC tissues of patients, and CAF-EVs were extracted and identified. EdU, colony formation, wound healing, and Transwell assays were performed. The OCSCC cells before and after CAF-EVs treatment were injected into mice to probe the effects of CAF-EVs on tumor growth and lymph node metastasis, respectively. The effect of CAF-EVs treatment on transcriptome changes in OCSCC cells was analyzed. Clinical data of patients with OCSCC were analyzed to determine the prognostic significance of the selected genes. Finally, loss-of-function assays were conducted to corroborate the involvement of polycomb complex protein BMI-1 (BMI1) and integrin beta1 (ITGB1).

**Results:**

CAF-EVs promoted the malignant behavior of OCSCC cells and accelerated tumor growth and lymph node metastasis in mice. CAF-EVs significantly increased the expression of BMI1 and ITGB1, and the expression of BMI1 and ITGB1 was negatively correlated with the overall survival and relapse-free survival of OCSCC patients. Knockdown of BMI1 or ITGB1 in OCSCC cells abated the promoting effects of CAF-EVs in vitro and in vivo.

**Conclusion:**

CAF-EVs elicited the metastasis-promoting properties in OCSCC by elevating BMI1 and ITGB1, suggesting that BMI1 and ITGB1 could be potential biomarkers and therapeutic targets for OCSCC.

**Supplementary Information:**

The online version contains supplementary material available at 10.1186/s12885-024-11855-0.

## Background

Oral cavity cancer is categorized under head and neck cancer and holds the sixteenth position among all malignancies worldwide, and more than 90% of oral cavity cancer originates from the squamous tissues, hence known as oral cavity squamous cell carcinoma (OCSCC) [1]. OCSCC remains a lethal disease exhibiting rising incidence, predominantly in younger patients, and global data of over 300,000 new diagnoses are presented each year [2]. In addition, the 5-year survival rate for OCSCC patients stands at only 47–66% as most cases were detected at a late stage of malignancy, and the rate of OCSCC recurrence ranged from 18 to 76% [3]. OCSCC is principally treated by combinations of surgery, radiotherapy, and chemotherapy, while the effect of traditional treatment methods is limited [4]. Therefore, it is urgent to identify potential biomarkers and therapeutic targets for improving the survival of affected patients.

Extracellular vesicles (EVs), membrane-derived vesicles secreted by different cells, can carry DNA, RNA, protein, microRNA, and other substances, thereby participating in the development of OCSCC [5]. Cancer-associated fibroblasts (CAF) are one of the principal components in the tumor microenvironment (TME), and CAF-derived EVs regulate malignant and non-malignant cells of the TME, thereby manipulating tumor progression, metastasis, and chemoresistance [6, 7]. Even though it has been proven that CAF-EVs significantly induced migration and invasion of OCSCC cells and enhanced a disseminated pattern of HSC-3 cell invasion in the 3D organotypic assay [8], in vivo evidence is still lacking. As for the cargoes of CAF-EVs, most concentration has been focused on microRNAs [9, 10], and we performed RNA-sequencing (RNA-seq) in the present study to screen for differentially expressed genes in OCSCC cells treated with CAF-EVs. Polycomb complex protein BMI-1 (BMI1) and integrin beta1 (ITGB1) were revealed to be two significantly upregulated mRNAs in OCSCC cells and predicted dismal overall survival (OS) and relapse-free survival (RFS) for patients with OCSCC. BMI1, a member of PRC1 that binds to the RING2/RING1b subunit to form a functional E3 ubiquitin ligase, was found to be overexpressed in various tumors, including endometrial cancer, laryngeal carcinoma, prostate cancer, and hepatocellular carcinoma [11]. ITGB1 was significantly upregulated or downregulated in solid cancers with controversial prognostic value [12]. However, the effects of BMI1 and ITGB1 encapsulated by CAF-EVs on OCSCC cell malignant phenotype remain unclear. Herein, we aimed to investigate the phenotypic alterations of OCSCC cells co-cultured with CAF-EVs and to substantiate the involvement of BMI1 and ITGB1.

## Methods

### Patients and specimens

All subjects gave informed consent before they participated in the study. The study was conducted following the *Declaration of Helsinki*, and the protocol was approved by the Ethics Committee and Institutional Review Board of the Stomatological Hospital of Guizhou Medical University. Thirty OCSCC patients without lymph node metastasis and 30 OCSCC patients with lymph node metastasis who underwent surgery at the Stomatological Hospital of Guizhou Medical University from 2017 to 2020 were included. The tumor and adjacent tissues obtained were stored in a -80 °C refrigerator. A three-year follow-up of the patients was documented after surgery.

CAF were extracted from the tumor tissues of four OCSCC patients who underwent surgical resection at the Stomatological Hospital of Guizhou Medical University in 2022. Tumor samples were washed three times with PBS immediately. CAF were isolated from tumor samples by magnetically activated cell sorting using antibodies against cell type-specific markers. In brief, tumors were dissociated into single cells using collagenase. Undesired cell types were removed using Dynabeads (Invitrogen™) Magnetic Cell Separation Technology based on cell markers, including CD45 (lymphocytes), CD68 (macrophages), CD31 (endothelial cells), and Ep-CAM (epithelial cells). CAF were then isolated by positive selection based on THY1 (fibroblasts). CAF were cultured in DMEM containing 10% FBS with 1% penicillin/streptomycin at 37 °C with 5% CO_2_.

### Cell cultures and treatment

Normal oral epithelial cells (CP-H203), human oral mucosal fibroblasts (CP-H205, named normal fibroblasts, NFs thereafter), and OCSCC cell lines SCC-25 (CL-0569) and CAL-27 (CL-0265) were purchased from Procell (Wuhan, Hubei, China) and cultured using DMEM containing 10% FBS with 1% penicillin/streptomycin at 37 °C with 5% CO_2_.

### Isolation and identification of CAF-EVs

CAFs were incubated in FBS-depleted DMEM for 24 h. Subsequently, the medium was collected and centrifuged at 2000 × *g* for 30 min to remove dead cells, cellular debris, and large-sized EVs and obtain a conditioned medium. Total Exosome Isolation Reagent (4478359, Thermo Fisher Scientific Inc., Waltham, MA, USA) was supplemented to the conditioned medium, and the solution was incubated at 2 to 8 °C overnight. EVs were separated by centrifugation at 10,000 × *g* for 60 min, followed by resuspension of the precipitate in PBS.

The morphology of CAF-EVs was observed by transmission electron microscopy (TEM). Approximately 30 µL of the prepared CAF-EVs solution was loaded onto a 2 mm diameter carbon-coated copper mesh and allowed to adsorb for 10 min. Samples were fixed with 10 µL of 2.5% glutaraldehyde for 5 min, rinsed once with distilled water, stained with 1% uranyl acetate, and dried for 20 min at room temperature. The prepared CAF-EVs were then observed under the TEM (JEM-2100, Hitachi, Tokyo, Japan) at 160.0 kV, and images were taken with a digital camera.

Nanoparticle tracking analysis (NTA) was performed using a Nano Sight NS300 instrument (Nano Sight, Malvine, UK) configured with a blue (488 nm) laser and sCMOS camera to determine particle size and concentration. Samples (stored at -80 °C) were thawed, gently vortexed, and then diluted in PBS to achieve particle concentrations consistent with the optimal analytical range of the software (2 × 10^8^ to 1.8 × 10^9^ particles per milliliter).

### Western blot analysis

CAFs, NFs, and CAF-EVs were lysed using RIPA-containing lysis buffer (R0278-50ML, Sigma-Aldrich) on ice after which the supernatant was collected by centrifugation at 12,000 *g*. BCA Protein Quantification Kit (23229, Thermo Fisher) was used to quantify protein content. Proteins were denatured in a water bath, separated by sodium dodecyl sulfate-polyacrylamide gel electrophoresis, and transferred to a polyvinylidene fluoride membrane. The membranes were sealed with 5% goat serum (16210072, Thermo Fisher) for 1 h and incubated at 4 °C overnight with specific primary antibodies to CD81 (1:700, ab109201, Abcam, Cambridge, UK), TSG101 (1:20000, ab125011, Abcam), Flotillin 1 (1:1000, ab133497, Abcam), ALIX (1:1000, ab275377, Abcam), Calnexin (1:1000, AF2425, Beyotime Biotechnology Co., Ltd., Shanghai, China), Vimentin (1:2000, 10366-1-AP, ProteinTech Group, Chicago, IL, USA), FAP (1:1000, ab314456, Abcam), α-SMA (1:2000, 14395-1-AP, ProteinTech Group), GAPDH (1:2000, AF1186, Beyotime), BMI1 (1:2000, 10832-1-AP, ProteinTech Group), ITGB1 (1:2000, 12594-1-AP, ProteinTech Group). The membranes were incubated with goat anti-rabbit horseradish peroxidase-coupled secondary antibody (1:2000, 7074, Cell Signaling Technologies, Beverly, MA, USA) at room temperature for 1 h. Protein bands were visualized by chemiluminescence, and Image J was used to analyze the gray value of the bands. The quantitative analyses were standardized according to the internal reference GAPDH.

### Fluorescent labeling of EVs and tracing EVs uptake by OCSCC cells

CAF-EVs (50 µg) were incubated with lipophilic tracer Dil solution (D282, Thermo Fisher) for 20 min at 37 °C. Excess Dil was removed by centrifugation. Labeling efficiency was analyzed using a fluorometer. Dil-labeled CAF-EVs (50 µg) were added to the medium of SCC-25 and CAL-27 cells and treated at 37 °C for 3 h. Recipient cells were fixed with 4% paraformaldehyde for 10 min and permeabilized with 0.1% Triton X-100 (HFH10, Thermo Fisher) for 5 min (both at room temperature). OCSCC cells were stained with DAPI for 30 min at room temperature, mounted with Prolong® Diamond Anti-Quenching Reagent (P36975, Thermo Fisher), and covered with coverslips. The cells were visualized by fluorescence microscopy. CAF-EVs not labeled with Dil (50 µg) were also used in subsequent functional assays.

### Lentiviral vector infection

After culturing to 50% confluence, the OCSCC cells were infected with lentivirus-encapsulated knockdown BMI1 plasmid or knockdown ITGB1 plasmid (both from VectorBuilder, Guangzhou, Guangdong, China). After 12 h of infection at 37 °C in 5% CO_2_, the medium was replaced with fresh medium and positive cells were screened using 2 µg/mL puromycin. The intervention effect of the target genes was subsequently analyzed using RT-qPCR.

### Cell proliferation assay and colony formation assays

OCSCC cell proliferation was analyzed using the Click-iT™ EdU Cell Proliferation Imaging Kit (Alexa Fluor™ 647) (C10340, Thermo Fisher). The cells were seeded into 6-well plates and incubated for 12 h before adding 10 µM EdU for 1 h at 37 °C in 5% CO_2_. After fixation using 4% paraformaldehyde, the cells were permeabilized with PBS containing 0.5% Triton X-100 at room temperature for 20 min and incubated with Click-iT ® reaction mixture with fluorescent labeling for 20 min. After labeling the nuclei with DAPI, the number of proliferating cells was observed under a fluorescence microscope, and the positive rate of EdU (%) was counted.

The cells were seeded into 6-well plates at 500 per well and incubated at 37 °C with 5% CO_2_ for two weeks, followed by fixation of the cells with 4% paraformaldehyde for 15 min and staining with crystal violet for 10 min. The number of cell colonies formed was counted under a light microscope.

### Cell migration and invasion assays

OCSCC cells were cultured on Petri dishes or plates. A scratch was made after the cells became a confluent monolayer, and the cellular debris was washed away. Images were taken at 0 and 24 h after scratching.

The cell suspension containing 10^4^ OCSCC cells (200 µL) was seeded into the apical chamber of 24-well plates coated with Matrigel (CLS354234, Sigma-Aldrich), and the basolateral chamber was supplemented with fresh medium containing FBS. After a 24-h incubation at 37 °C with 5% CO_2,_ the cells in the basolateral chamber were collected, fixed using 4% paraformaldehyde for 15 min, and stained with 1% crystal violet for 15 min. The number of invaded cells was observed and counted under the microscope. For the determination of cell migration, the addition of Matrigel was not required, and the cell suspension was seeded directly into the apical chamber.

### Tumorigenesis and metastasis assay in vivo

This study was approved by the Institutional Animal Care and Use Committee of the Stomatological Hospital of Guizhou Medical University. The report of animal experiments is following the ARRIVE guidelines. BALB/c nude mice aged 4–6 weeks were procured from Vital River (Beijing, China). The nude mice were kept in a specific pathogen-free room with free access to adequate food and drinking water. After one week of acclimatization, 30 nude mice were then divided into 5 groups of 6 mice each: the control, CAF-EVs, NC knockdown, BMI1 knockdown, and ITGB1 knockdown groups. After the indicated treatments, the concentration of SCC-25 cells was adjusted to 1 × 10^6^ cells/mL of single-cell suspension and 0.2 mL of cell suspension was injected into the nude mice. The tumor volume of the nude mice was measured at five-day intervals after the injection of tumor cells.

For the lymph node metastasis assay, the tongue mucosa of each mouse was injected with 0.2 mL of SCC-25 cell suspension after indicated treatments (1 × 10^6^ cells/mL). Tumor and lymph node volume (mm^3^) = width (mm)^2^ × length (mm)/2. The mice were euthanized by intraperitoneal injection of pentobarbital sodium (150 mg/kg) on day 30, and the tumors and lymph nodes were collected and weighed.

### Immunohistochemistry

Tumors and lymph nodes removed from nude mice were sectioned, dewaxed with xylene, and rehydrated with ethanol. After antigen retrieval in citrate buffer, endogenous peroxidase was removed from the sections using 3% hydrogen peroxide solution, and 5% goat serum was added for a 2-h sealing at room temperature. The sections were incubated overnight at 4 °C with the primary antibody to Ki67 (1:1000, ab15580, Abcam), followed by a 1-h incubation with goat anti-rabbit horseradish peroxidase coupled secondary antibody (1:200, ab6721, Abcam) at room temperature. After color development using DAB, hematoxylin counter-staining, ethanol dehydration, and xylene clearing, the sections were sealed with neutral gum to observe the cell positivity rate (%) under the microscope.

### Hematoxylin-Eosin (HE) staining

Paraffin-embedded sections of lymph nodes were subjected to xylene and gradient concentration of ethanol treatment and tumor cell infiltration in lymph node tissues was analyzed using the HE Staining Kit (E607318, Shanghai Sangon Biological Engineering Technology & Services Co., Ltd., Shanghai, China) according to the instructions of the kit. After dewaxing and rehydration, the sections were stained with hematoxylin for 5 min, treated with acetic acid, washed with ethanol, stained with eosin staining solution for 1 min, dehydrated with gradient ethanol, cleared with xylene, and sealed with neutral gum. The infiltration of tumor cells in the lymph nodes of nude mice was observed and confirmed by pathologists under a light microscope.

### RT-qPCR

Total RNA from OCSCC cells was extracted by TRIzol reagent and reverse transcribed to cDNA using SuperScript™ III Reverse Transcriptase (18080085, Thermo Fisher). RT-qPCR was then performed using PowerTrack™ SYBR Green Master Mix (A46012, Thermo Fisher) via a real-time PCR detection system. The fold changes amplification for targeted genes was normalized to the housekeeping gene β-actin respectively by the 2^−△△CT^ method. The sequences for BMI1 were 5’-GGTACTTCATTGATGCCACAACC-3’ (Sense), 5’-CTGGTCTTGTGAACTTGGACATC-3’ (antiSense), ITGB1, 5’-GGATTCTCCAGAAGGTGGTTTCG-3’ (Sense), 5’-TGCCACCAAGTTTCCCATCTCC-3’ (antiSense), PXN-AS1, 5’-GGAAGGCGCTGCCTCG-3’ (Sense), 5’-TTTGCGTGCTTCCTCTTTGC-3’ (antiSense), HISTH1B 5’-TTAAGCTGGGCCTCAAGAGC-3’ (Sense), 5’-GAGCCTTTGGGGCTTTGTTG-3’ (antiSense), LOC100128361, 5’-AACGACATTCCCTCCGGAAC-3’ (Sense), 5’-TGTCTGTAAAGTGGGACGCC-3’ (antiSense), β-actin 5’-CACCATTGGCAATGAGCGGTTC-3’ (Sense), 5’-AGGTCTTTGCGGATGTCCACGT-3’ (antiSense).

### Statistical analysis

All experiments were carried out in triplicate. The data are presented as the mean ± standard deviation (SD). Statistical analyses were carried out using GraphPad Prism 8.0.2 software (GraphPad, San Diego, CA, USA). Paired or unpaired *t-tests* were applied to compare data between two groups, and one-way or two-way ANOVA was used for multiple comparisons. Survival analysis was performed with a log-rank test. Values of *p* less than 0.05 were considered statistically significant.

## Results

### CAF-EVs induce the malignant behavior of OCSCC cells

**Fig. 1:**
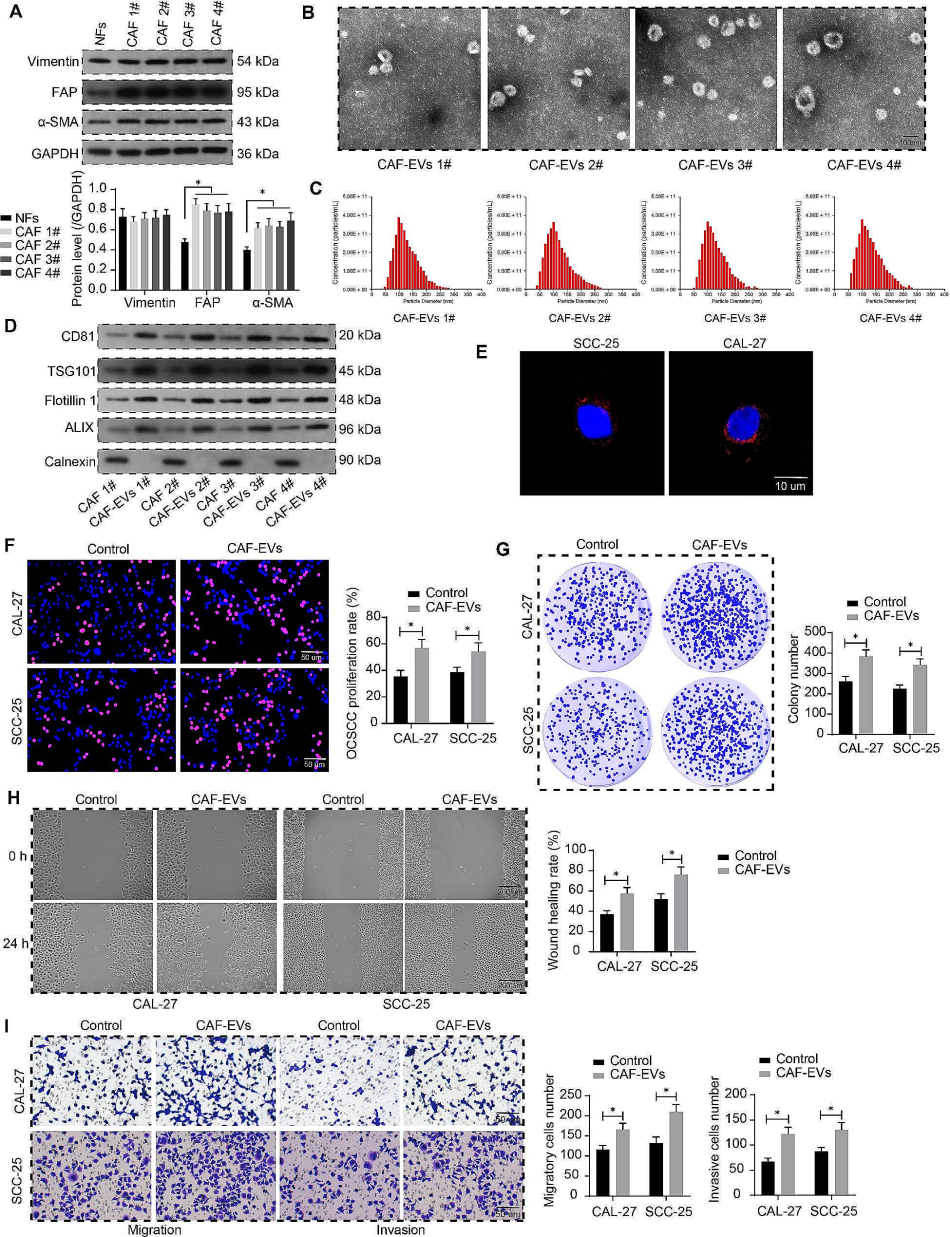
Characterization and effects of CAF-EVs. **A** Vimentin, FAP, and α-SMA protein expression in NF and CAF was examined using western blot analysis. **B** The morphology of CAF-EVs observed under a TEM. **C** The diameter size of CAF-EVs was analyzed using NTA. **D** CD81, TSG101, FLOT1, ALIX, and Calnexin in CAF and derived CAF-EVs were detected using western blot analysis. **E** The uptake of Dil-labeled CAF-EVs by OCSCC cells. **F** The effect of CAF-EVs on the proliferative capacity of OCSCC cells was measured using an EdU proliferation assay. **G** The effect of CAF-EVs on the colony-forming ability of OCSCC cells was measured using colony formation assays. **H** The wound healing rate of OCSCC cells before and after CAF-EVs treatment was determined using a wound healing assay. **I** The effect of CAF-EVs on the migration and invasion of OCSCC cells. Two-way ANOVA. Data are presented as means ± SD. Results are representative of at least three independent experiments (*p <.05)

We isolated CAF 1, 2, 3, 4# from fresh tumor tissues of four OCSCC patients. Both extracted CAF and purchased NF expressed Vimentin, as detected by western blot, but the expression of FAP and α-SMA in CAF was significantly higher than that in NFs, which correlates with the typical characteristics of CAF (Fig. 1A). TEM analysis was performed to observe the morphology of EVs, which were seen to be circular or cup-shaped bilayer structures of similar size and morphology (Fig. 1B). NTA was also conducted to detect the diameter size of the EVs, and the diameter of the EVs was concentrated at 50–200 nm (Fig. 1C). EVs were enriched with the marker proteins CD81, TSG101, Flotillin 1, and ALIX, and did not express the negative marker Calnexin. By contrast, CAF expressed all proteins (Fig. 1D). CAL-27 and SCC-25 cells were treated with the mixture of four CAF-EVs (0.05 µg EVs/10^3^ cells). Moreover, the uptake of EVs by CAL-27 and SCC-25 cells was examined using 50 µg Dil-labeled CAF-EVs, confirming the successful uptake of EVs by the cells (Fig. 1E). CAF-EVs not labeled with Dil (50 µg) were used in subsequent functional assays.

EdU proliferation assay was performed to analyze the effect of CAF-EVs on the proliferative ability of OCSCC cells, and CAF-EVs greatly promoted the proliferation of OCSCC cells (Fig. 1F). Colony formation assay confirmed that CAF-EVs enhanced the colony formation ability of OCSCC cells (Fig. 1G). Wound healing assays were carried out to analyze the migration of OCSCC cells, and OCSCC cells after CAF-EVs treatment accelerated the wound healing process (Fig. 1H). CAF-EVs as demonstrated by Transwell assay enhanced the migration and invasion of OCSCC cells (Fig. 1I).

### CAF-EVs exacerbate tumor formation and lymph node metastasis of OCSCC cells

**Fig. 2:**
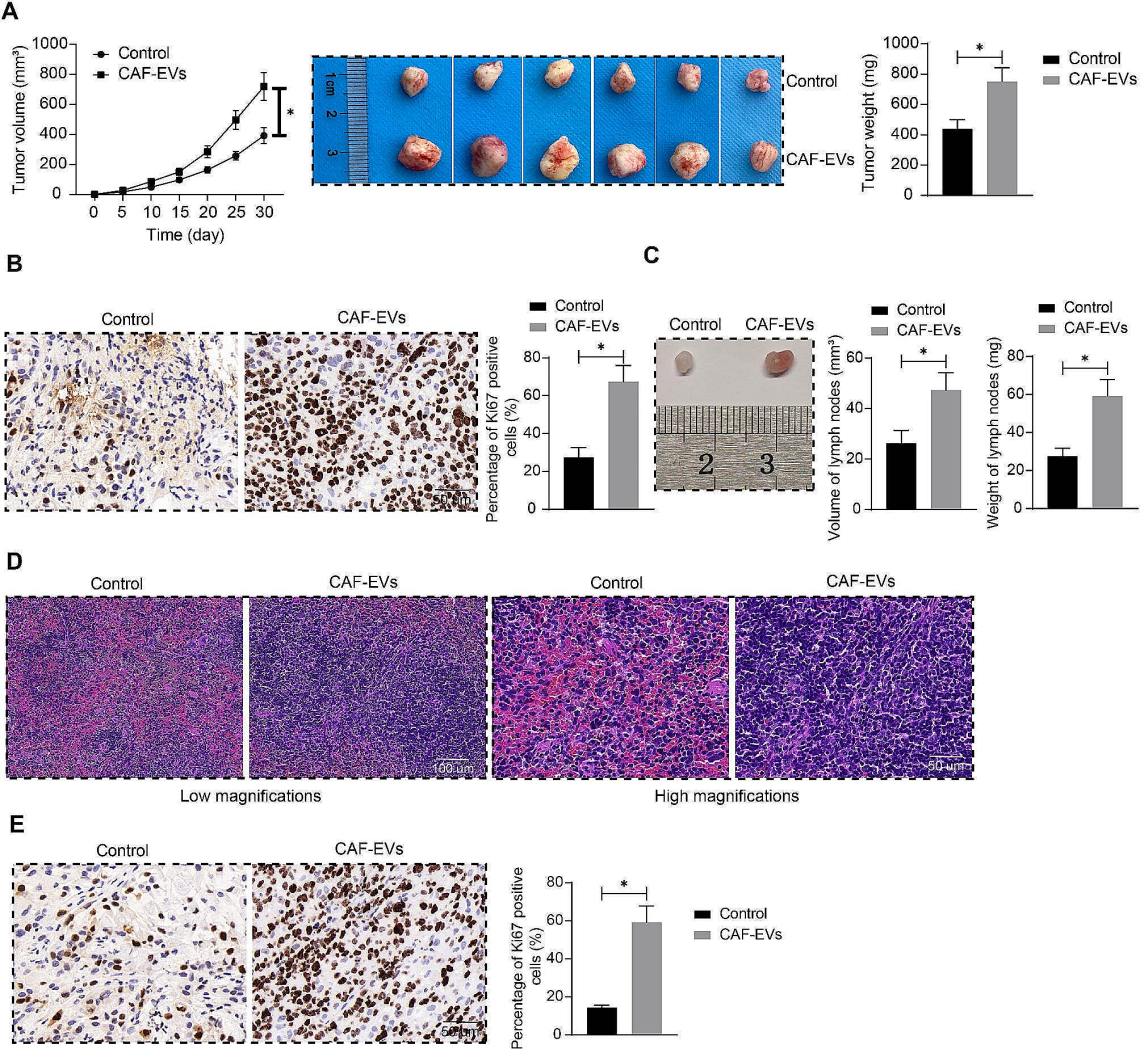
CAF-EVs exacerbate tumor formation and lymph node metastasis in tumor-bearing mice. **A** The weight and volume of tumors formed by SCC-25 cells treated with or without CAF-EVs (*n* = 6). **B** Ki67 expression in tumors determined by immunohistochemistry (*n* = 6). **C** The weight and volume of lymph nodes in mice induced with in vivo orthotopic xenograft model (*n* = 6). **D** Distribution of SCC-25 in lymph nodes observed by HE staining (*n* = 6). **E** Determination of Ki67 expression in lymph nodes by immunohistochemistry (*n* = 6). Two-way ANOVA or unpaired t-test. Data are presented as means ± SD (*p <.05)

SCC-25 cells treated with or without CAF-EVs were injected subcutaneously into nude mice to establish xenograft tumors, and tumor growth was observed for 30 d, during which tumor volume was evaluated at 5-d intervals (Fig. 2A). Xenograft tumors established from SCC-25 cells treated with CAF-EVs grew faster and yielded larger tumor burden in nude mice. Ki67 expression in tumors was determined by immunohistochemistry (Fig. 2B), and the percentage of Ki67-positive cells in tumors increased after treatment with CAF-EVs. SCC-25 cells treated or not with CAF-EVs were injected into the tongue mucosa of nude mice. After 30 days, lymph nodes were collected to measure the volume and weighed, and lymph nodes of nude mice injected with CAF-EVs-treated SCC-25 were larger in size and heavier in weight (Fig. 2C). HE staining was performed to observe the distribution of SCC-25 in the lymph nodes, and SCC-25 cells were more densely distributed in the lymph nodes after CAF-EVs treatment (Fig. 2D). The expression of Ki67 in lymph nodes was determined by immunohistochemistry, and a greater proportion of Ki67-positive cells were found in the lymph nodes of nude mice injected with CAF-EVs-treated SCC-25 (Fig. 2E).

### BMI1 and ITGB1 are possible cargoes of CAF-EVs affecting OCSCC cells

**Fig. 3:**
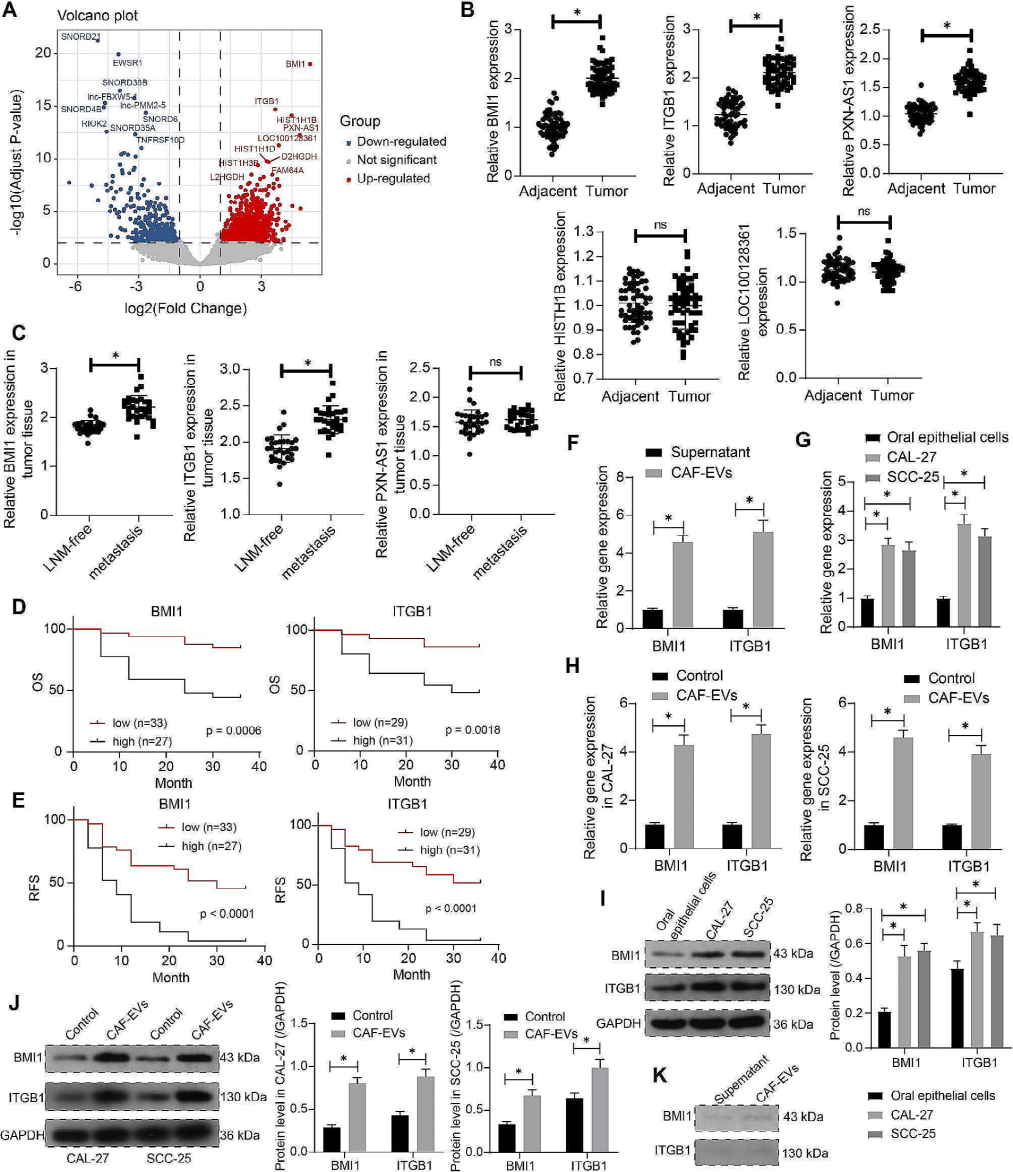
BMI1 and ITGB1 are essential for the phenotypic modulation of OCSCC cells by CAF-EVs. **A** Differentially expressed genes in OCSCC cells treated with or without CAF-EVs filtered through RNA-sequencing. **B** The expression of five genes in OCSCC tissues and adjacent tissues from OCSCC patients was detected by RT-qPCR. **C** The expression of BMI1, ITGB1, and PXN-AS1 in the tumor tissues of OCSCC patients with or without lymph node metastasis. **D** The correlation between BMI1 and ITGB1 expression and OS in OCSCC patients was analyzed using a rank log-test. **E** The correlation between BMI1 and ITGB1 expression and RFS in OCSCC patients was analyzed using a rank log-test. **F** BMI1 and ITGB1 expression in CAF-EVs and the supernatant were measured using RT-qPCR. **G** CAL-27 and SCC-25 cells, with normal oral epithelial cells as control, was measured using RT-qPCR. **H** The expression of BMI1 and ITGB1 in CAL-27 and SCC-25 cells before and after CAF-EVs treatment was measured using RT-qPCR. **I** The protein expression of BMI1 and ITGB1 in normal oral epithelial cells and OCSCC cells was measured using western blot analysis. **J** The protein expression of BMI1 and ITGB1 in CAL-27 and SCC-25 cells before and after CAF-EVs treatment was measured using western blot analysis. **K**. The protein expression of BMI1 and ITGB1 carried by CAF-EVs was measured using western blot analysis. Paired or unpaired t-test, two-way ANOVA. Data are presented as means ± SD. Results are representative of at least three independent experiments (*p <.05)

The effect of CAF-EVs treatment on transcriptome expression in OCSCC cells was analyzed by RNA-seq (performed on Illumina Hiseq 2500 platform). Differential expression analysis was performed using the R software (v.4.2.2) package “DESeq2” (v.1.40.0), with *p*-values corrected by fdr (BH) to filter out low abundance and outliers. The significance thresholds were set at adjusted *p-*value < 0.01, and the absolute value of fold change > 2. Visualization was completed using the R package “ggpubr” (v0.4.0) (Fig. 3A). The top five genes (BMI1, ITGB1, HISTH1B, PXN-AS1, and LOC100128361) whose expression was significantly elevated after treatment with CAF-EVs were selected for clinical analysis. OCSCC and adjacent tissues were collected from 30 patients with lymph node metastasis and 30 patients without lymph node metastasis. The expression of BMI1, ITGB1, HISTH1B, PXN-AS1, and LOC100128361 in OCSCC tissues and adjacent tissues of 60 patients was detected by RT-qPCR, in which the expression of BMI1, ITGB1, and PXN-AS1 was significantly elevated in tumor tissues (Fig. 3B). The expression of BMI1, ITGB1, and PXN-AS1 in 30 patients without lymph node metastasis was compared with 30 patients with lymph node metastasis, and only the expression of BMI1 and ITGB1 was further elevated in the tumor tissues of patients with lymph node metastasis (Fig. 3C). The correlation between the expression of BMI1 and ITGB1 and the prognosis of 60 OCSCC patients was further analyzed. The prognostic significance of high and low expression of BMI1 and ITGB1 on patients’ OS (Fig. 3D) and RFS (Fig. 3E) was analyzed. The higher the expression of BMI1 and ITGB1, the lower the OS and RFS.

We first found the enrichment of BMI1 and ITGB1 in the CAF-EVs relative to the supernatant (Fig. 3F). RT-qPCR was performed to examine the expression of BMI1 and ITGB1 in CAL-27 and SCC-25 cells, and normal oral epithelial cells were used as control. The expression of BMI1 and ITGB1 was significantly elevated in OCSCC cells compared to normal oral epithelial cells (Fig. 3G). The changes in BMI1 and ITGB1 expression in CAL-27 and SCC-25 cells before and after CAF-EVs treatment were detected by RT-qPCR, and the expression of BMI1 and ITGB1 in CAL-27 and SCC-25 cells were elevated after CAF-EVs treatment (Fig. 3H). The results of western blot experiments showed that the protein expression of BMI1 and ITGB1 was also significantly elevated in OCSCC cells (Fig. 3I) and CAF-EVs treatment further enhanced the protein expression of BMI1 and ITGB1 in OCSCC cells (Fig. 3J). However, no significant BMI1 and ITGB1 protein was detected in CAF-EVs (Fig. 3K), suggesting that CAF-EVs delivered BMI1 and ITGB1 mRNAs into OCSCC cells.

### CAF-EVs promote malignant aggressiveness of OCSCC cells dependent on BMI1 and ITGB1

**Fig. 4:**
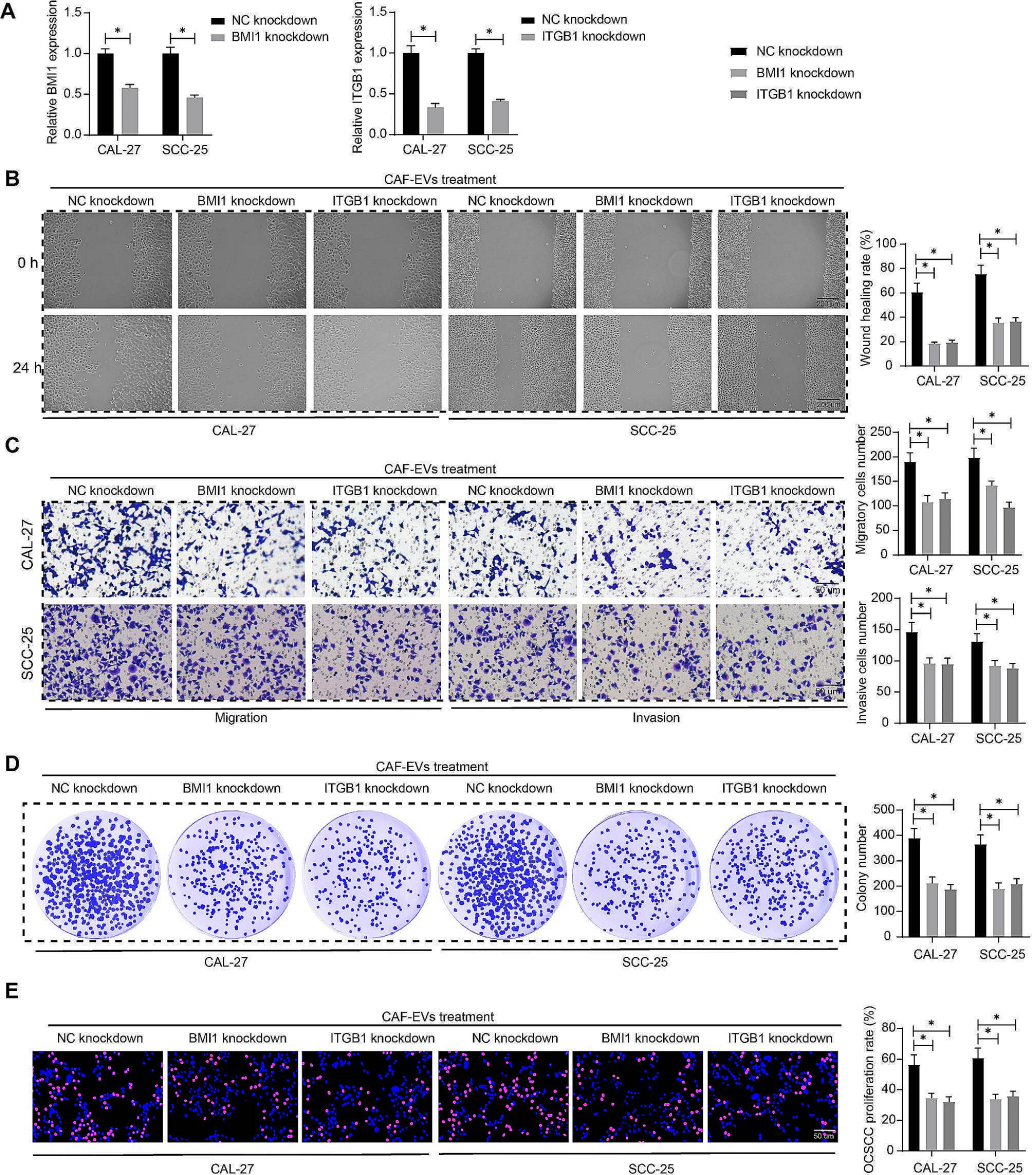
Knockdown of BMI1 and ITGB1 abates the promoting effects of CAF-EVs on OCSCC cells. **A** The expression of BMI1 and ITGB1 in OCSCC cells with BMI1 or ITGB1 knockdown was measured using RT-qPCR. B The wound healing rate of OCSCC cells with BMI1 or ITGB1 knockdown was determined using a wound healing assay. **C** The effect of BMI1 or ITGB1 knockdown on the migration and invasion of OCSCC cells. **D** The effect of BMI1 or ITGB1 knockdown on the proliferative capacity of OCSCC cells measured using EdU proliferation assay. **E** The effect of BMI1 or ITGB1 knockdown on the colony-forming ability of OCSCC cells was measured using colony formation assay. Two-way ANOVA. Data are presented as means ± SD. Results are representative of at least three independent experiments (*p <.05)

OCSCC cells with stable knockdown of BMI1 and ITGB1 were constructed by lentiviral infection and co-cultured with CAF-EVs, respectively. Firstly, we examined the expression of BMI1 and ITGB1 in OCSCC cells using RT-qPCR and revealed that the knockdown was effective (Fig. 4A). Wound healing assay was performed to analyze the wound healing rate of OCSCC cells, and the migratory ability of OCSCC cells was decreased after knockdown of BMI1 or ITGB1 (Fig. 4B). Transwell assay was performed to compare the migratory and invasive activities of OCSCC cells with knockdown of BMI1 and ITGB1 after CAF-EVs treatment, and both migratory and invasive abilities of OCSCC cells were repressed after BMI1 and ITGB1 knockdown (Fig. 4C). Finally, we observed that the knockdown of BMI1 or ITGB1 led to reduced colony formation and EdU-positive cells in OCSCC cells even in the presence of CAF-EVs (Fig. 4D, E).

### Reduced expression of BMI1 or ITBG1 slowed tumor formation and lymph node metastasis of OCSCC cells

**Fig. 5:**
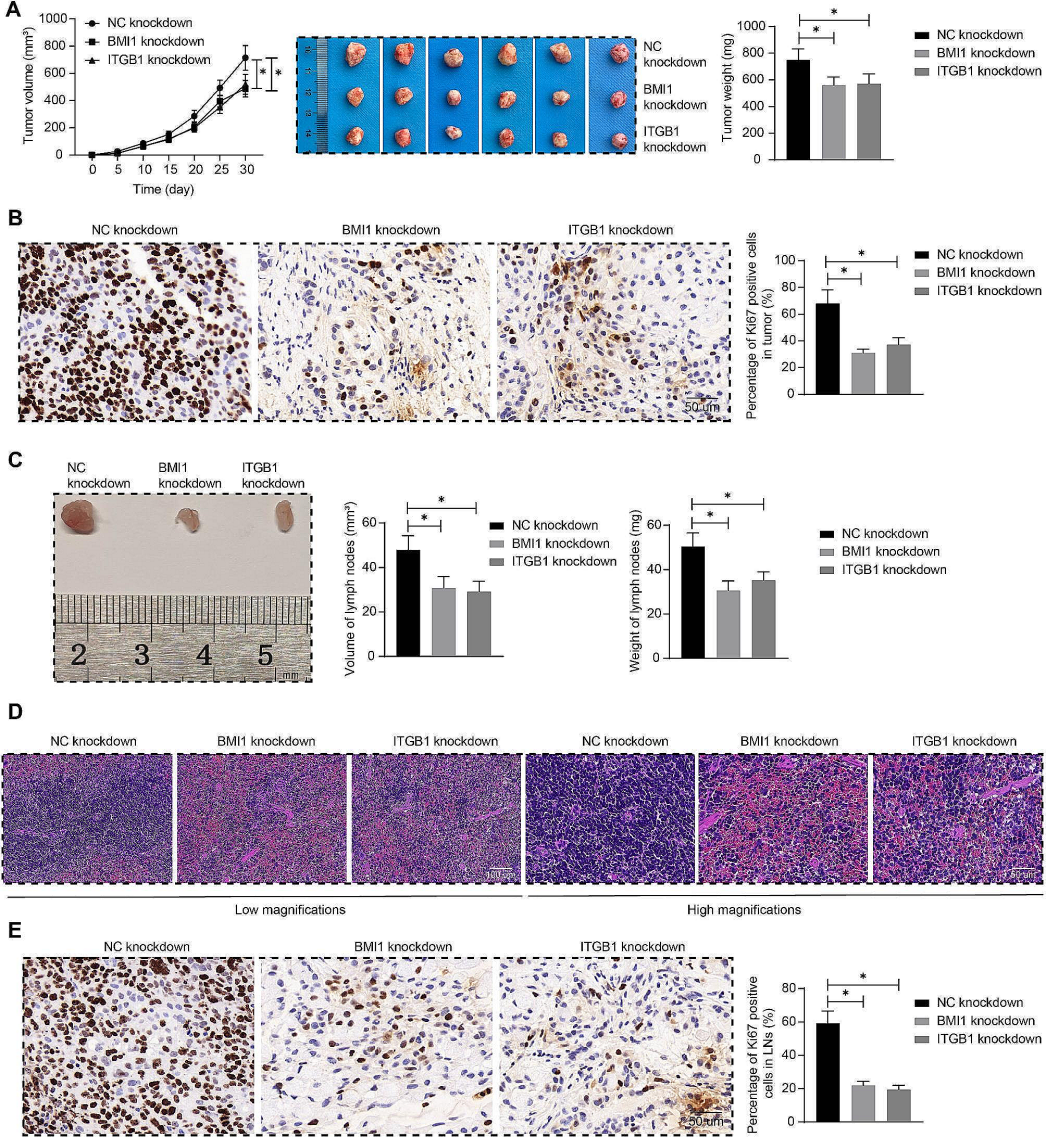
Knockdown of BMI1 and ITGB1 abates the tumor-promoting and metastasis-promoting properties of OCSCC cells in vivo. **A** The weight and volume of tumors formed by SCC-25 cells treated with BMI1 or ITGB1 knockdown (*n* = 6). **B** Ki67 expression in tumors determined by immunohistochemistry (*n* = 6). **C** The weight and volume of lymph nodes in mice induced with in vivo orthotopic xenograft model (*n* = 6). **D** Distribution of SCC-25 in lymph nodes observed by HE staining (*n* = 6). **E** Determination of Ki67 expression in lymph nodes by immunohistochemistry (*n* = 6). One-way or two-way ANOVA. Data are presented as means ± SD (*p <.05)

SCC-25 cells treated with CAF-EVs and SCC-25 cells with BMI1 and ITGB1 knockdown were injected subcutaneously into the back of mice to establish xenograft tumors, respectively. Tumors were weighed after euthanasia of nude mice on day 30, and slowed down the growth of OCSCC xenograft tumors after knockdown of BMI1 or ITGB1 was observed (Fig. 5A). Ki67 expression in tumors was determined by immunohistochemistry, and the proportion of Ki67-positive cells in tumors was decreased in tumor-bearing mice established by OCSCC cells with BMI1 or ITGB1 knockdown (Fig. 5B).

SCC-25 cells treated with CAF-EVs and SCC-25 cells knocked down with BMI1 and ITGB1 were injected into the tongue mucosa of mice, respectively. After 30 days, the mice were euthanized, and their lymph nodes were collected for weighing (Fig. 5C). As expected, BMI1 and ITGB1 knockdown reduced the weight and volume of lymph nodes. Furthermore, HE staining was performed to observe the distribution of OCSCC cells in lymph nodes (Fig. 5D), and immunohistochemistry was used to determine the expression of Ki67 in lymph nodes (Fig. 5E). The results showed that knockdown of BMI1 and ITGB1 reduced OCSCC cells in lymph nodes, and the proportion of Ki67-positive cells was decreased as well.

## Discussion

**Fig. 6:**
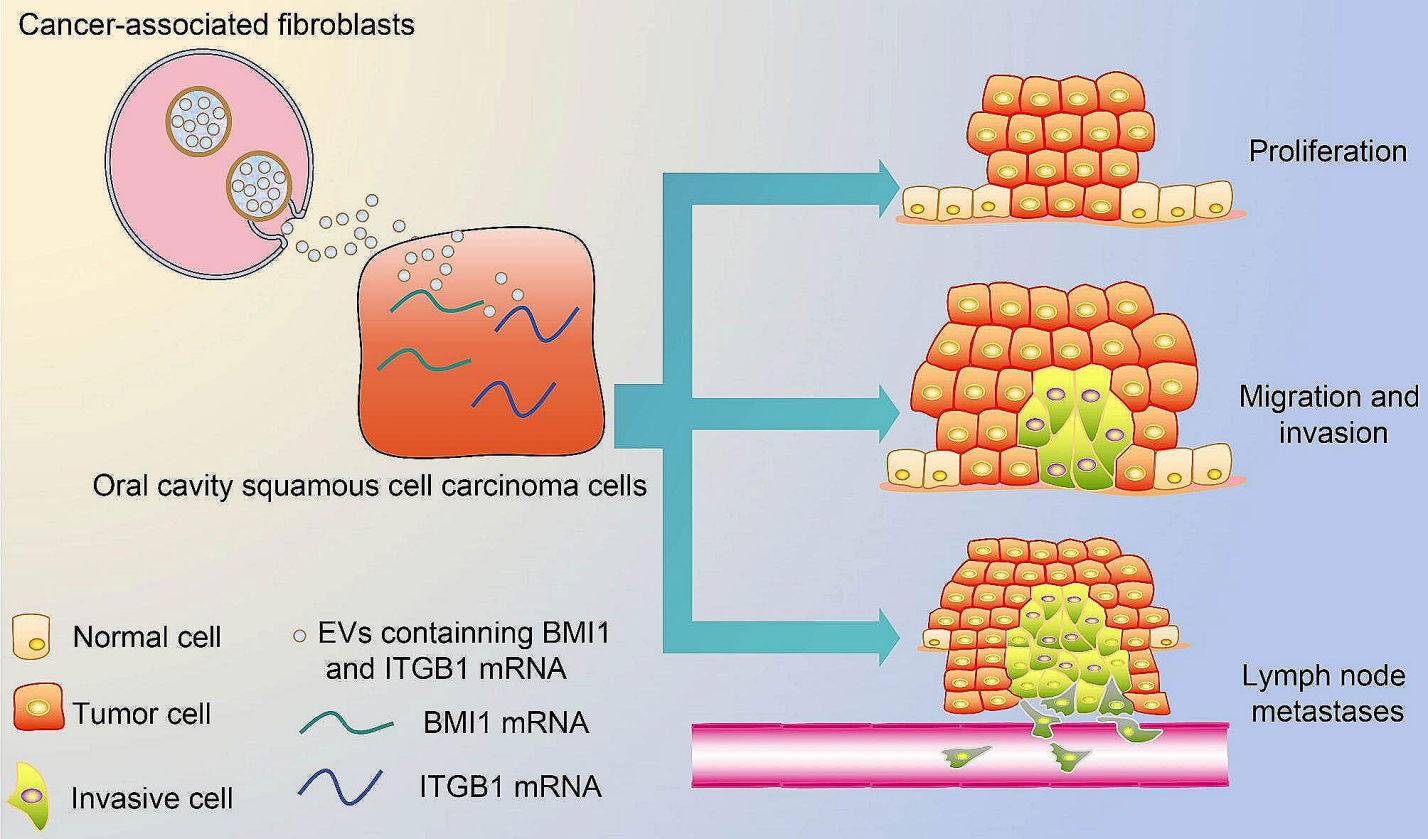
Diagram of the hypothetical mechanism clarifying the effect of BMI1 and ITGB1 in CAF-EVs on lymph node metastasis in OCSCC

CAF are accountable for increasing malignant features of head and neck cancer cells, and one of the many avenues taken by CAF to affect cancer cell behavior is the secretion of EVs with cargoes [13]. Lymph node metastases are common in many cancers and associated with unsatisfactory prognosis, and the formation of lymph node pre-metastatic niches is aided by the discharge of EVs [14]. Therefore, further investigation of the effect of EVs on lymph node metastases appears crucial. In this study, we investigated the role of CAF-derived EVs enriched with BMI1 and ITGB1 in modulating lymph node metastases in OCSCC, and our data suggested that CAF-derived EVs promoted lymph node metastases in OCSCC via delivering BMI1 and ITGB1 (Fig. 6).

The presence of increased CAF in the stroma of OCSCC predicted a decrease in overall survival, and the high presence of CAF was associated with parameters that worsen the prognosis in OCSCC, including advanced tumor, node, metastases classification, lymphatic and neural invasion, as well as extranodal metastatic spread [15]. EVs can be generally classified according to their size and origin: exosomes (50–200 nm), microvesicles (100–1000 nm), and apoptotic bodies (50-4000 nm) and have been reported to contain several types of specific surface markers, such as tetraspanins (CD81), MVB synthesis proteins (ALIX and TSG101), and membrane transporters and fusion proteins (FLOT1) [16]. In the study here, we observed that the extracted CAF-EVs showed these typical marker proteins and were in line with the reported diameter and morphology, indicating the successful extraction of CAF-EVs. Chernova et al. reported that exposure of healthy mesothelial cells to EVs derived from CAF resulted in pro-oncogenic signaling pathway activation and increased proliferation and motility [17]. CAF-EVs have also been revealed to induce the progression of various cancers, including colorectal cancer [18], cervical squamous cell carcinoma [19], and gastric cancer [20]. In the present study, we observed the enhancing effects of CAF-EVs on OCSCC cell migration, invasion, and colony formation. In addition, OCSCC cells co-cultured with CAF-EVs tended to form larger tumors in the xenograft-bearing mice and increased lymph node metastases in mice induced with an orthotopic xenograft model. The most recent report also suggested that a large number of CAFs were present in esophageal squamous cell carcinoma, leading to a poor prognosis, and CAF-derived EVs enhanced lymphangiogenesis in xenografts formed by esophageal SCC cells [21].

Subsequently, we identified BMI1 and ITGB1 as the cargoes of CAF-EVs to influence the OCSCC cells. BMI1 was revealed to be a prognostic indicator for brain tumors, lung adenocarcinoma, gastric cancer, renal cell carcinoma, colorectal cancer, and ovarian cancer [22]. Kim et al. showed that BMI1 was one of the four cancer stem cell-related genes in head and neck SCC that were associated with patient prognosis [23]. More relevantly, Kurihara et al. revealed that the protein and mRNA expression of BMI1 occurred during the invasion of patients with tongue SCC [24]. Moreover, elevated BMI1 is related to cervical node metastasis, high Ki-67 level, and shortened OS, and also acts as an independent prognostic factor for patients with tongue cancer [25]. METTL3 has been reported to promote OCSCC proliferation and metastasis through BMI1 m6A methylation [26]. All these findings suggested the association between BMI1 dysregulation and metastasis. Interestingly, the function of BMI1 in cholangiocarcinoma tumorigenesis and metastasis has been linked to EVs [27]. In the present study, we also found that the tumor-promoting and metastasis-promoting effects of CAF-EVs in vitro and in vivo were compromised by BMI1 knockdown. On the other hand, ITGB1 has been identified as an important gene for metastasis, progression, and prognosis for pancreatic cancer [28], lung adenocarcinoma [29], and gastric cancer [30]. Furthermore, Takahara et al. showed that SIPA1 promotes invasion and migration in OCSCC by regulating ITGB1 [31]. ALKBH5 has been recently reported to induce lymphatic metastasis in ovarian cancer by regulating ITGB1 expression, and antibodies that block ITGB1 are promising anti-metastatic agents [32]. In the present study, we found that CAF-EVs containing ITGB1 might be responsible for lymph node metastasis in OCSCC.

## Conclusion

In conclusion, this study demonstrated that the CAF-EVs can deliver oncogenic BMI1 and ITGB1, thus inducing lymph node metastasis in OCSCC. Considering that BMI1 and ITGB1 can also predict unsatisfactory outcomes for patients, these two mRNAs might be potential biomarkers and therapeutic targets for OCSCC patients.

### Electronic supplementary material

Below is the link to the electronic supplementary material.

Supplementary Material 1

Supplementary Material 2

## Data Availability

The datasets supporting the conclusions of this article are included within the article.
