# Supplementary material for: Cancer-associated fibroblast-derived extracellular vesicles promote lymph node metastases in oral cavity squamous cell carcinoma by encapsulating ITGB1 and BMI1
Source: BMC Cancer. 2024 Jan 22;24:113. doi: 10.1186/s12885-024-11855-0 (PMC10804601; doi:10.1186/s12885-024-11855-0)

Fig 1A: Vimentin, FAP, α-SMA, GAPDH

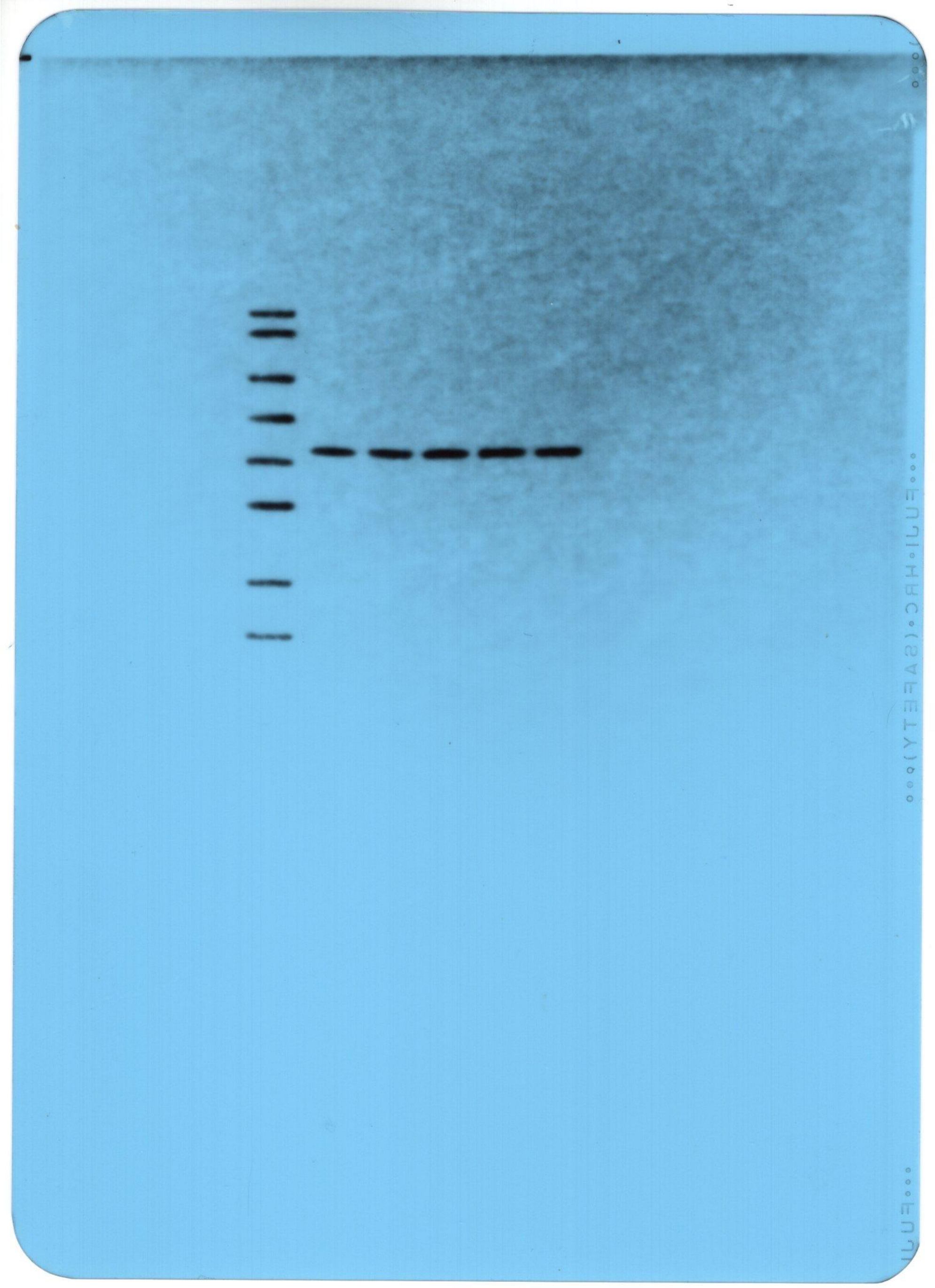

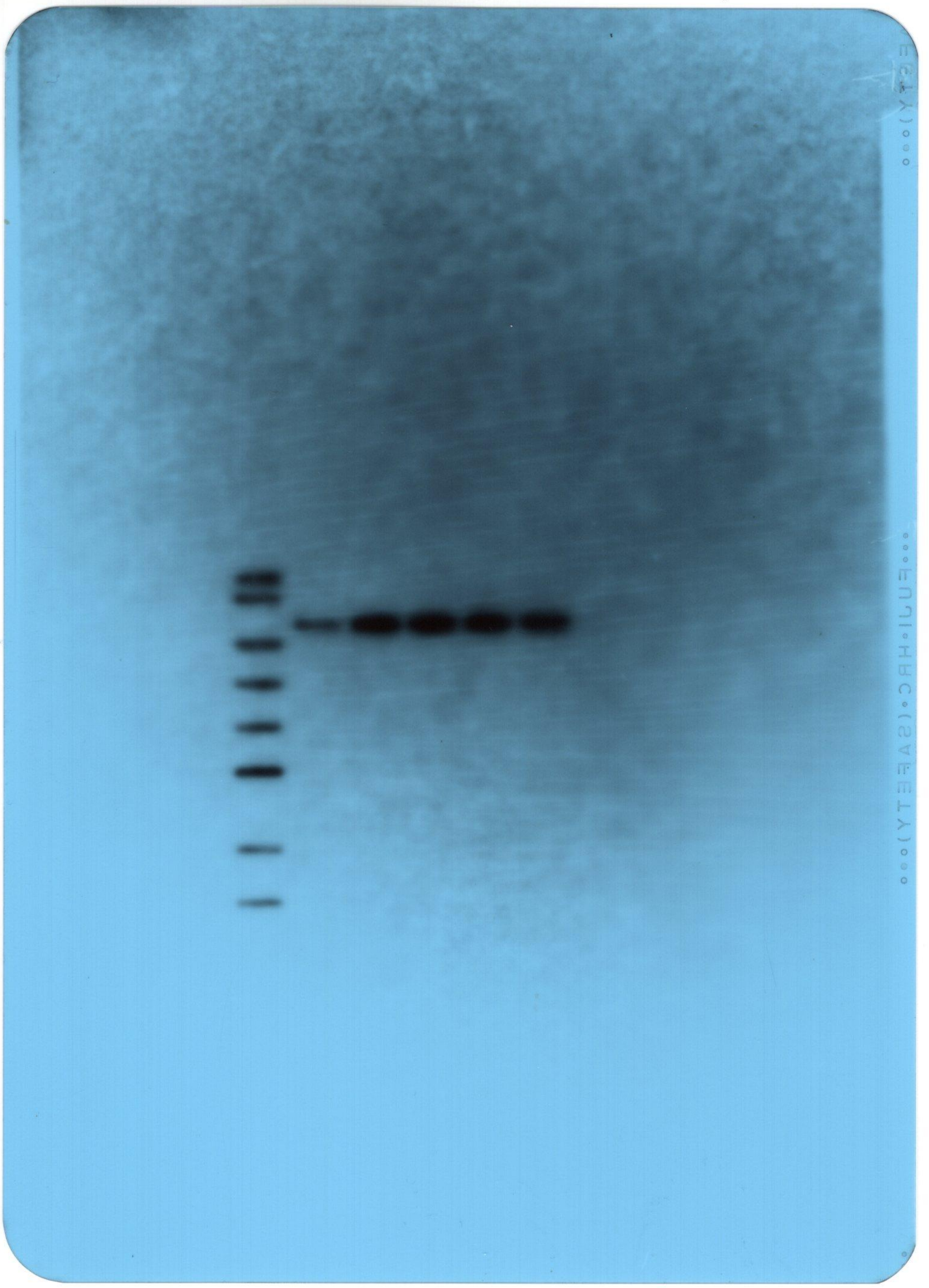

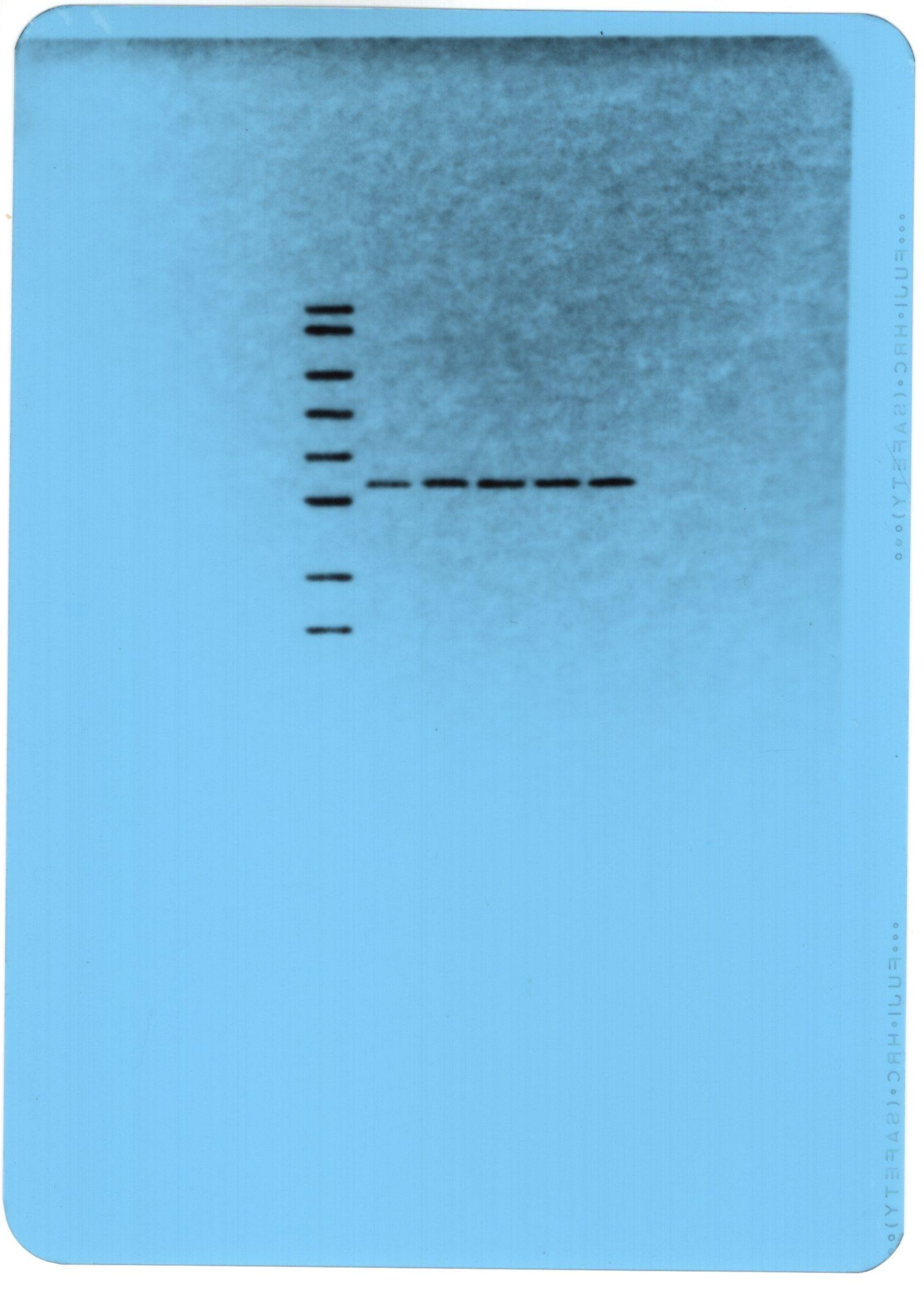

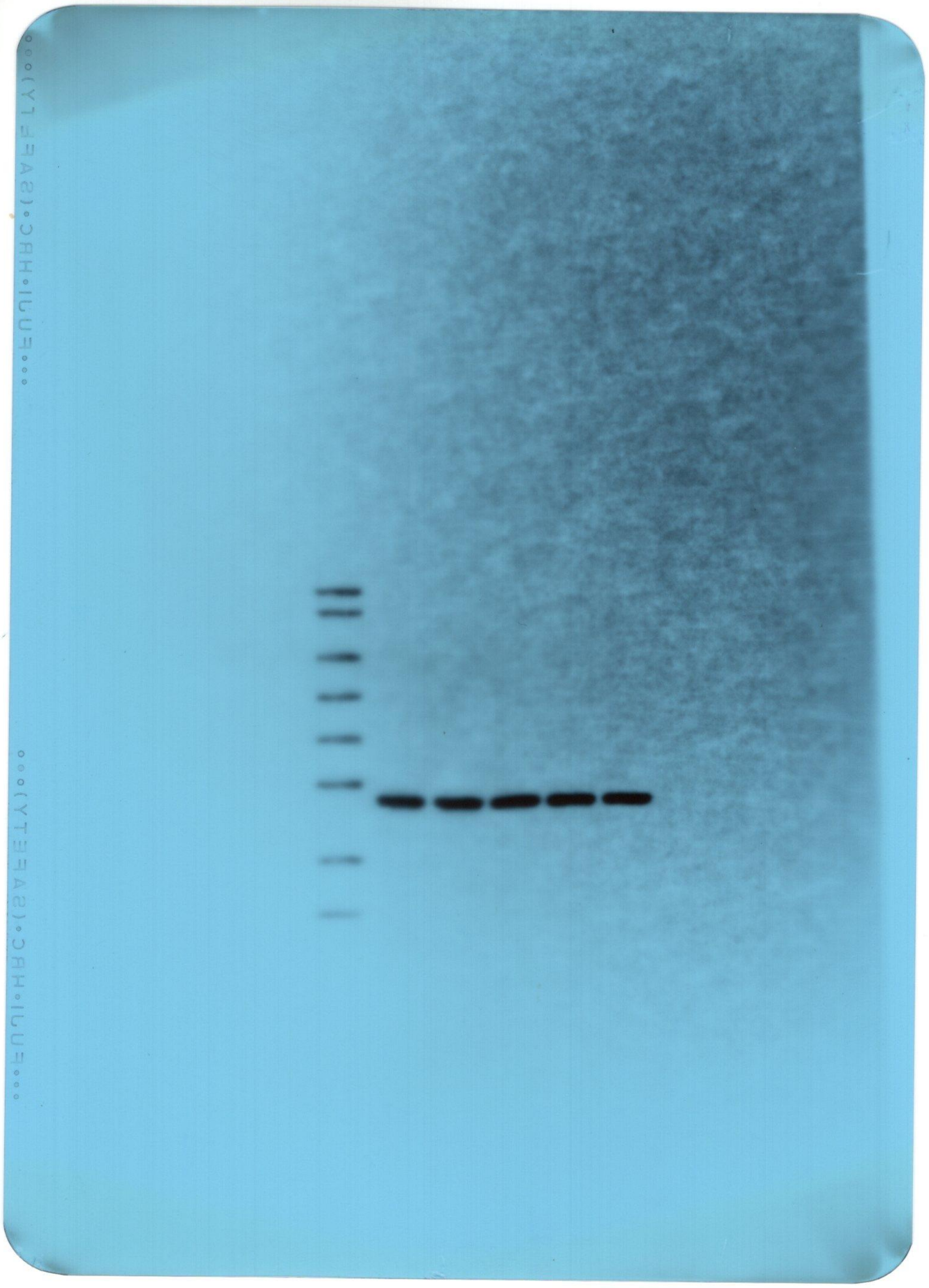

Fig 1D: CD81, TSG101, Flotillin 1, ALIX, Calnexin

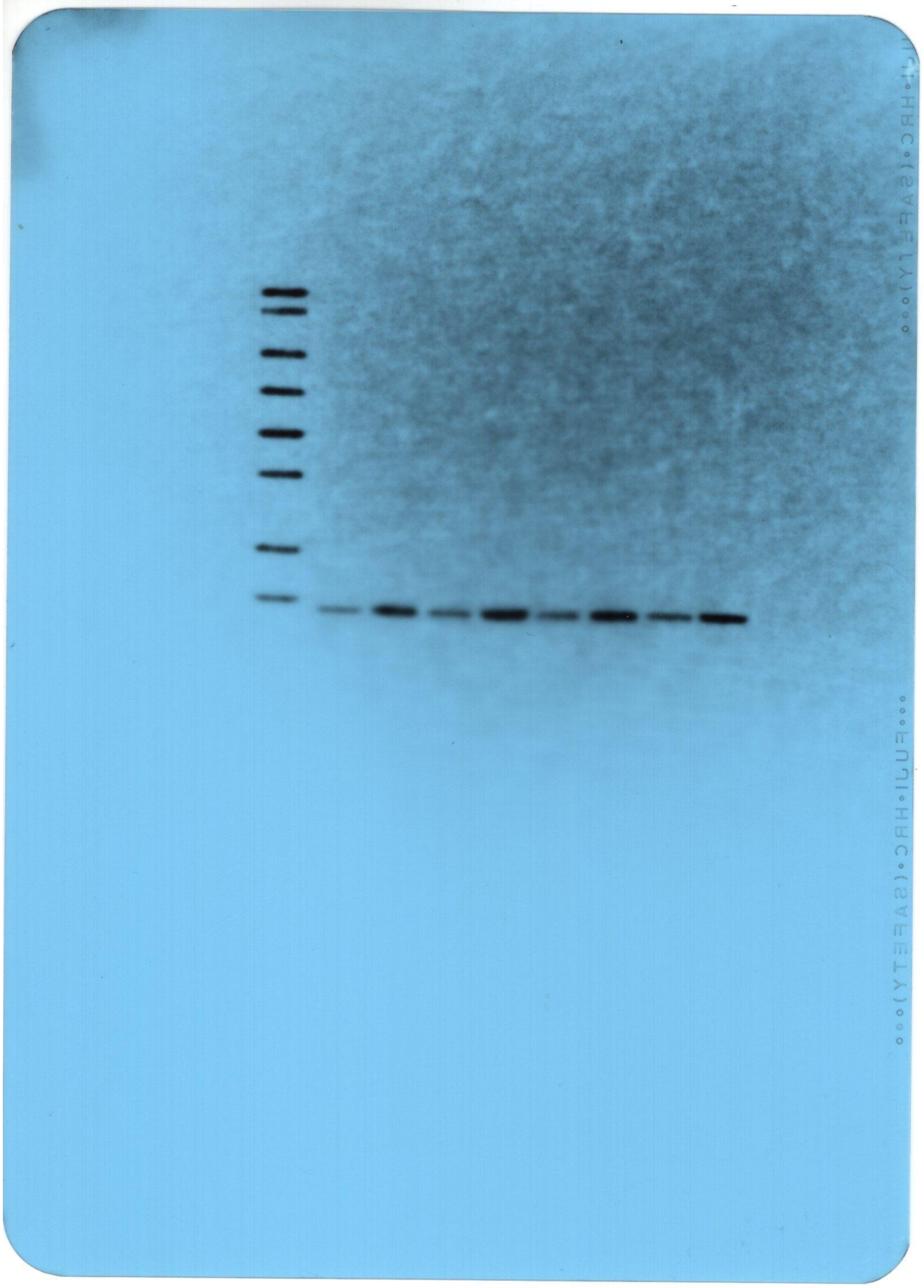

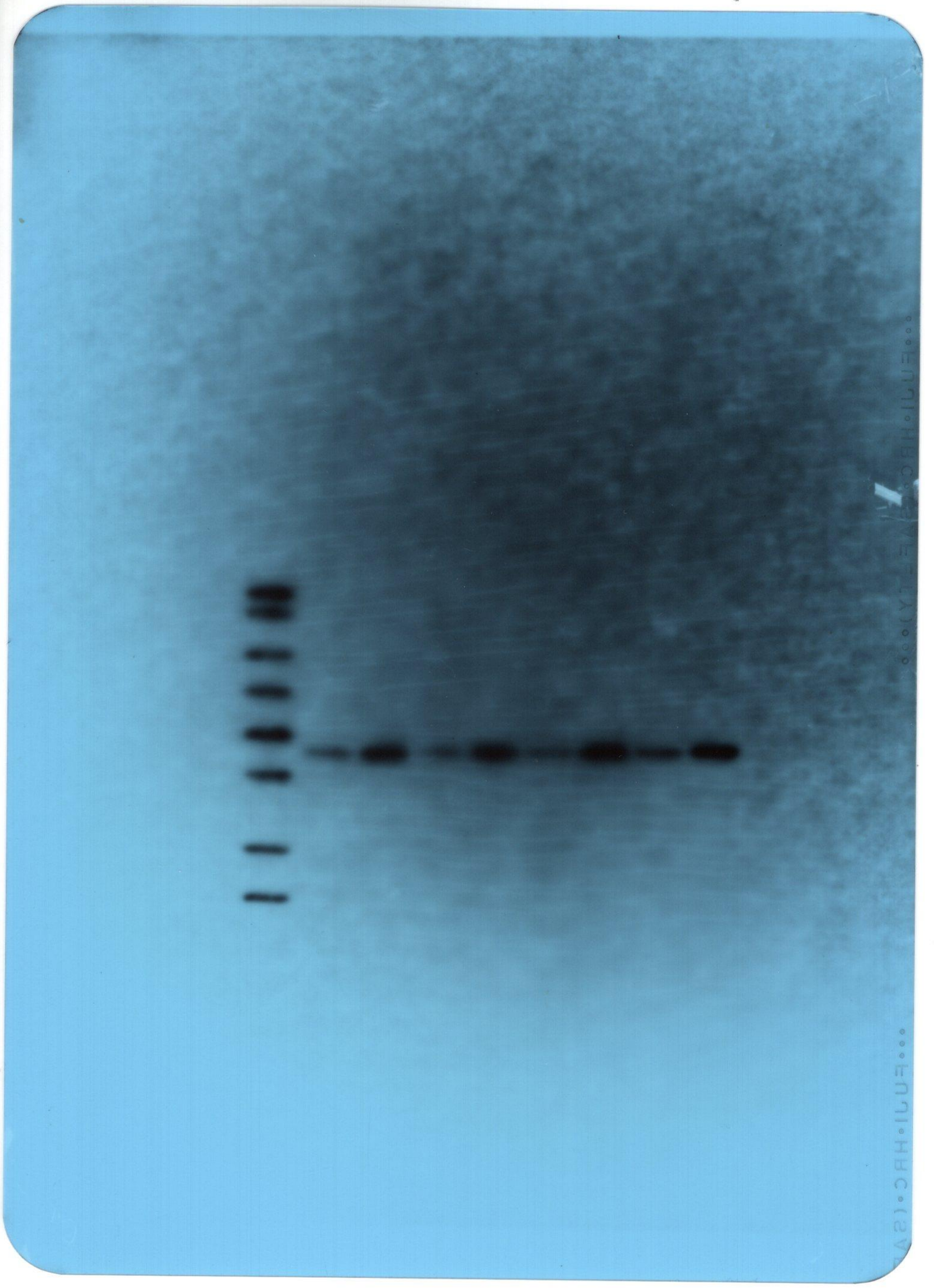

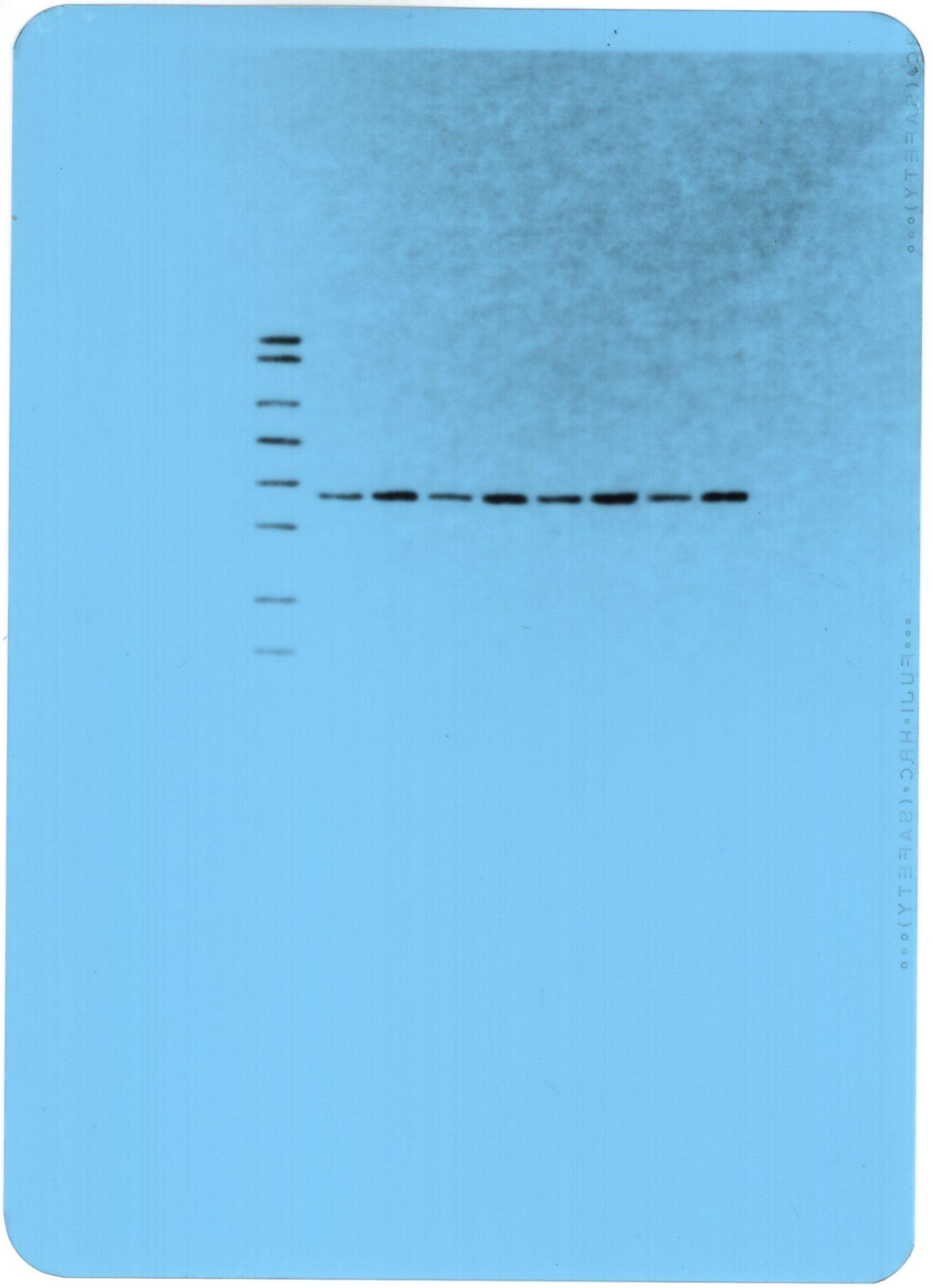

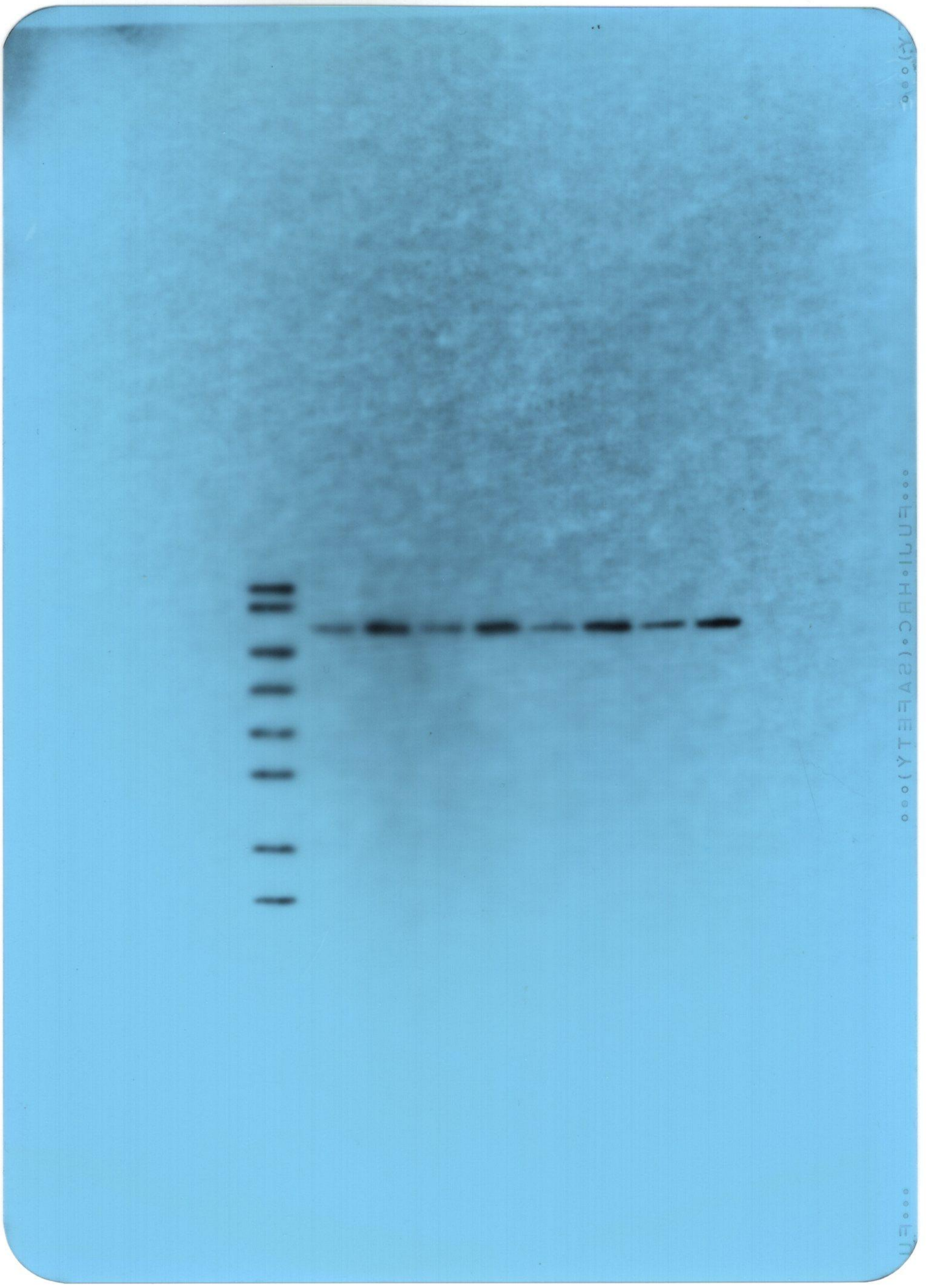

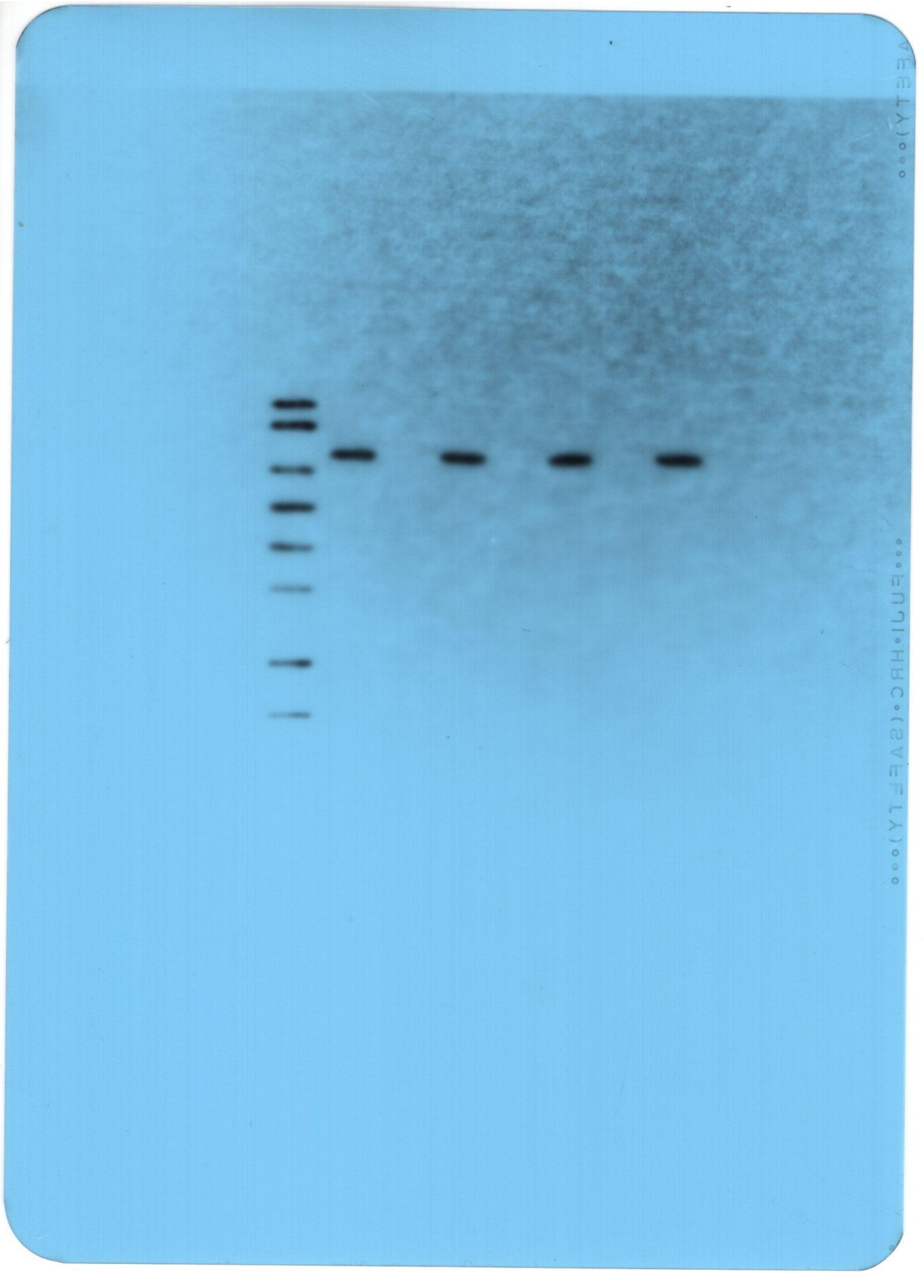

Fig 3I: BMI1, ITGB1, GAPDH

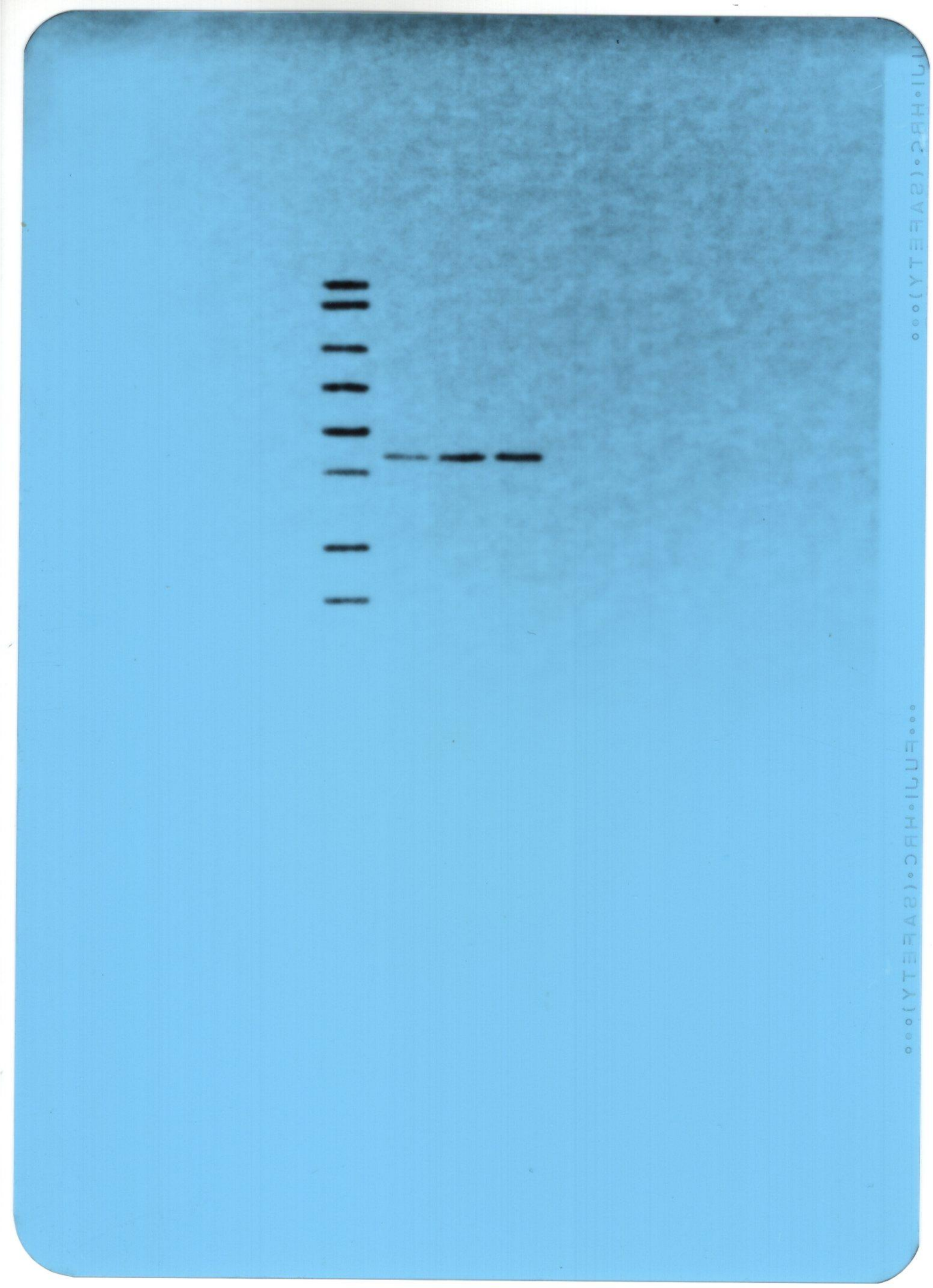

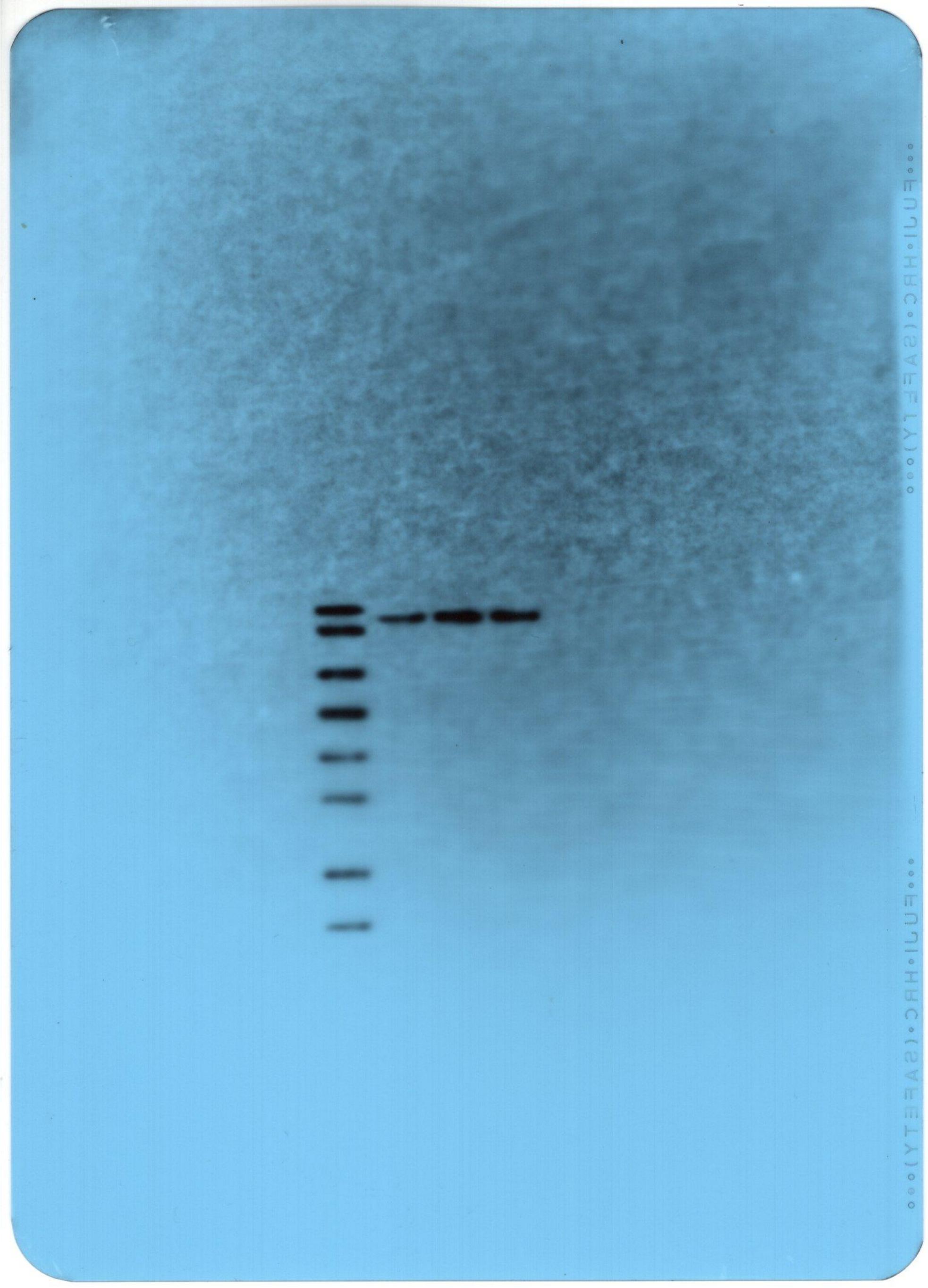

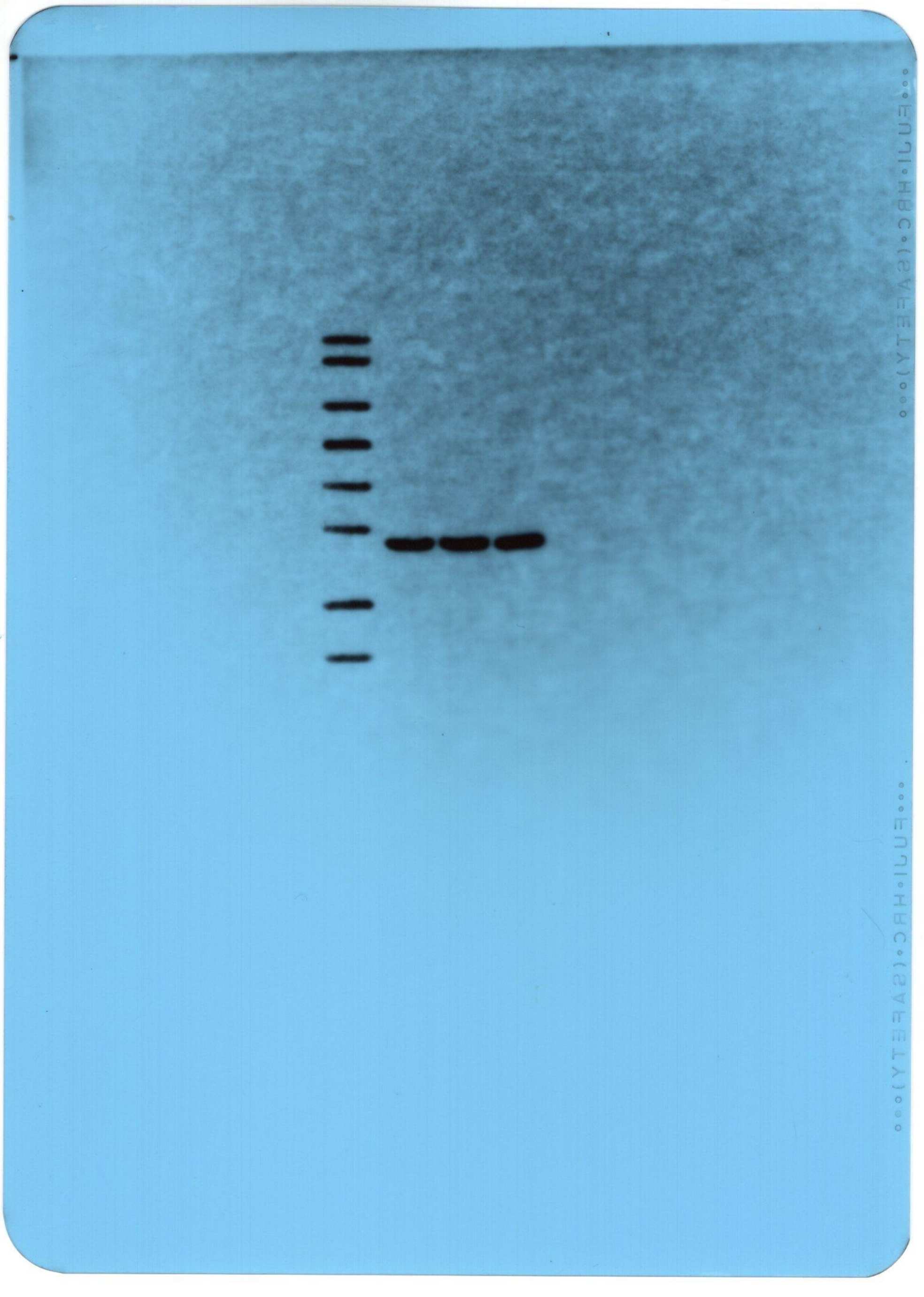

Fig 3J: BMI1, ITGB1, GAPDH

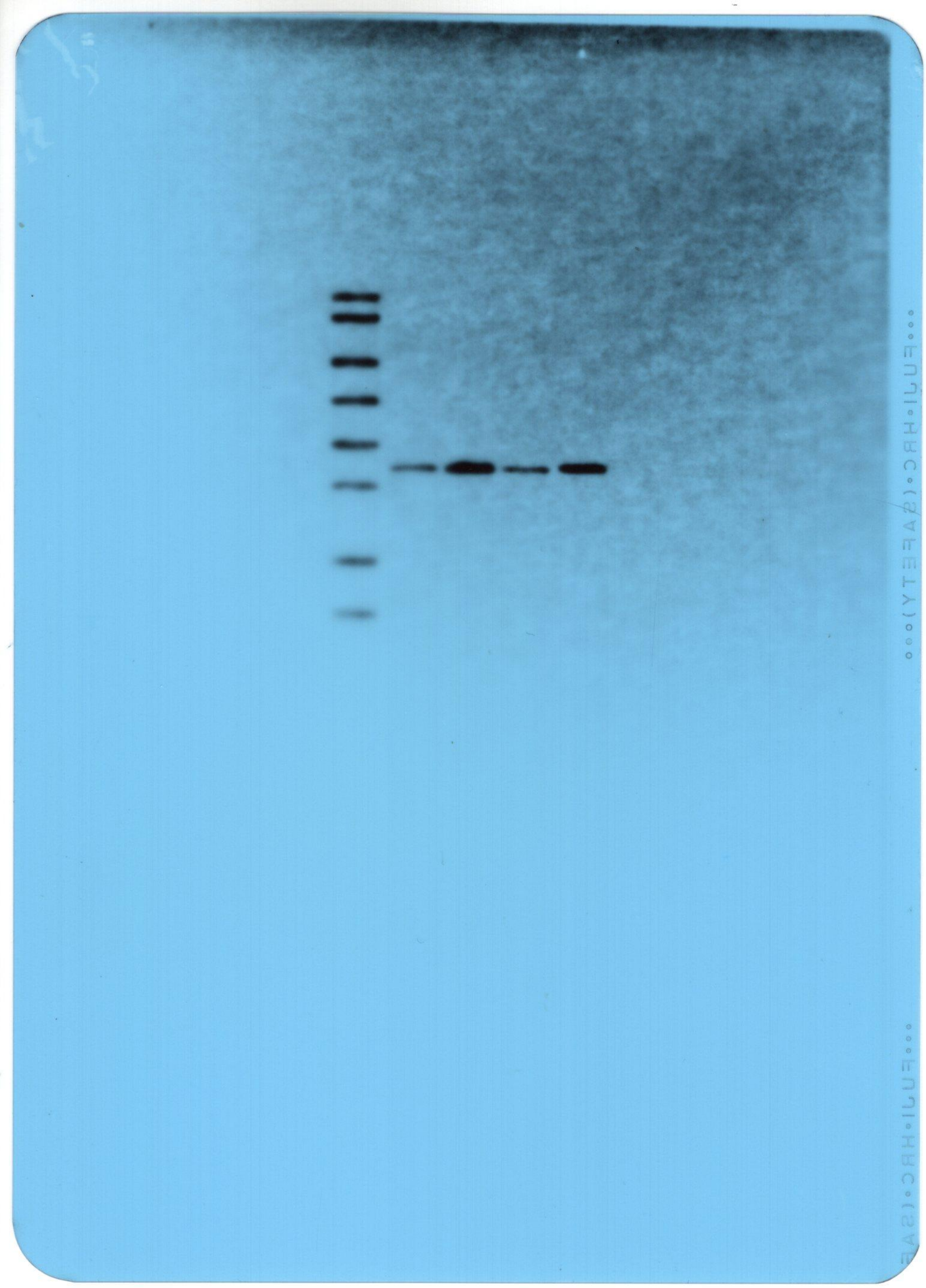

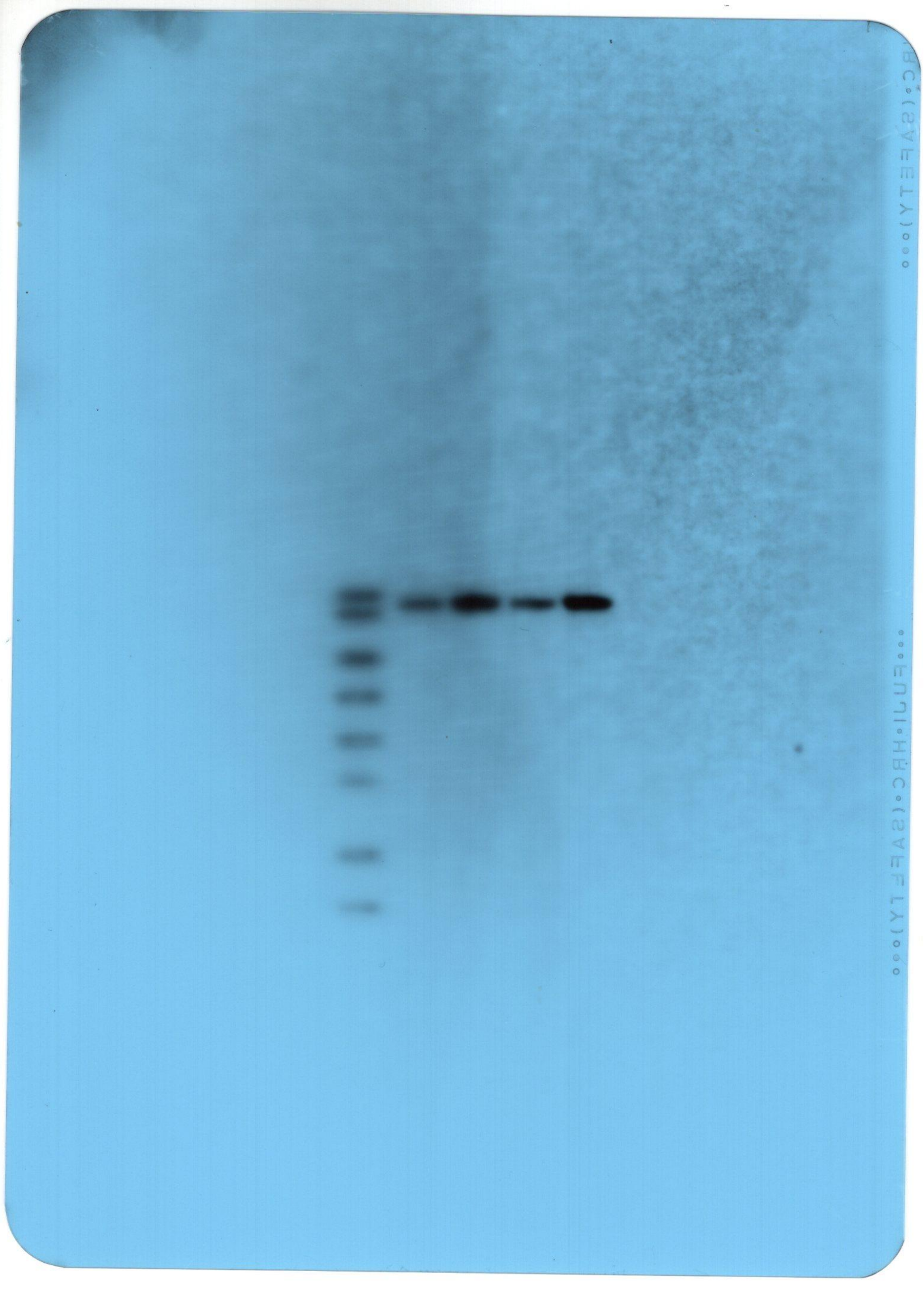

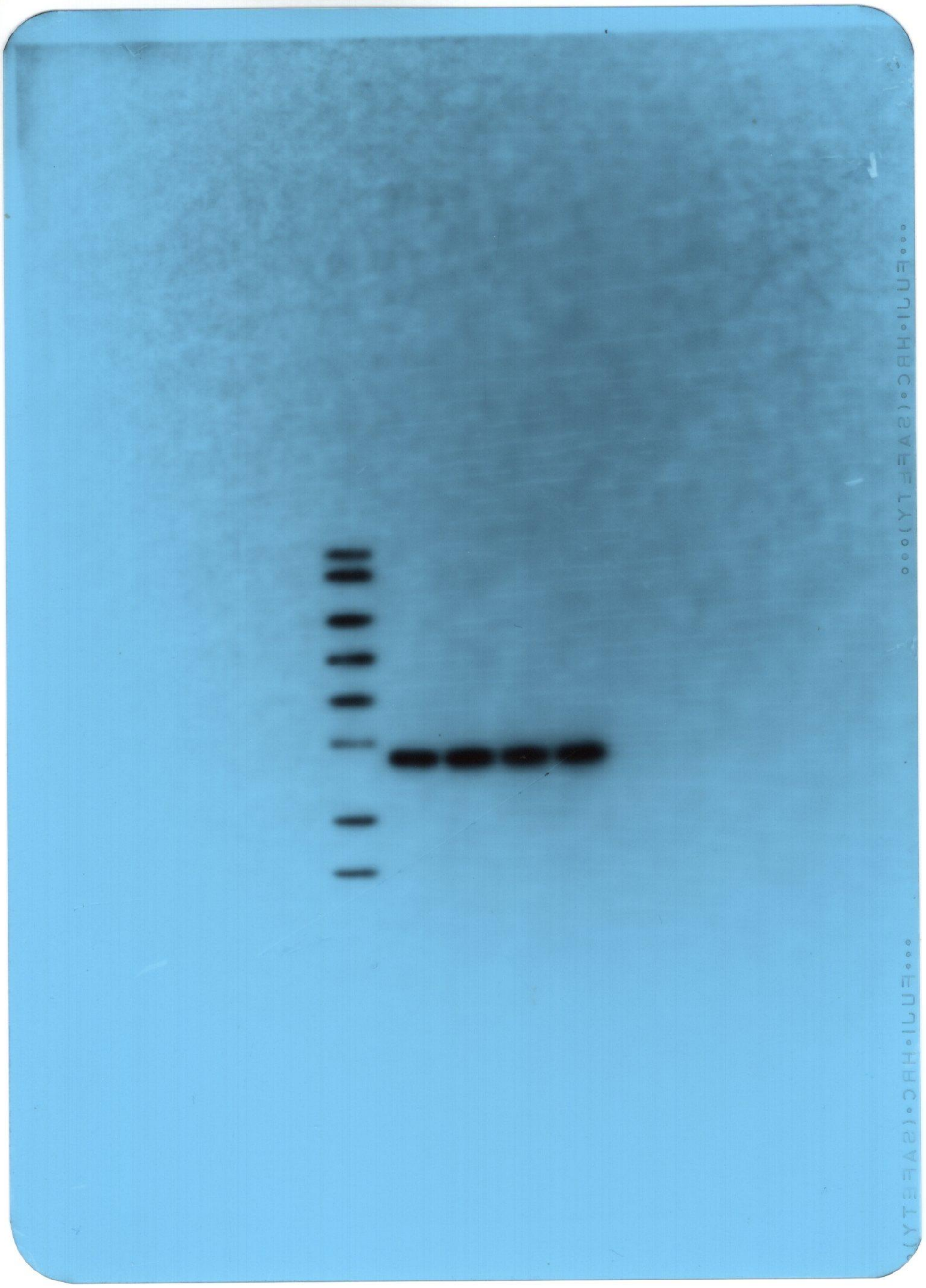

Fig 3K: BMI1, ITGB1

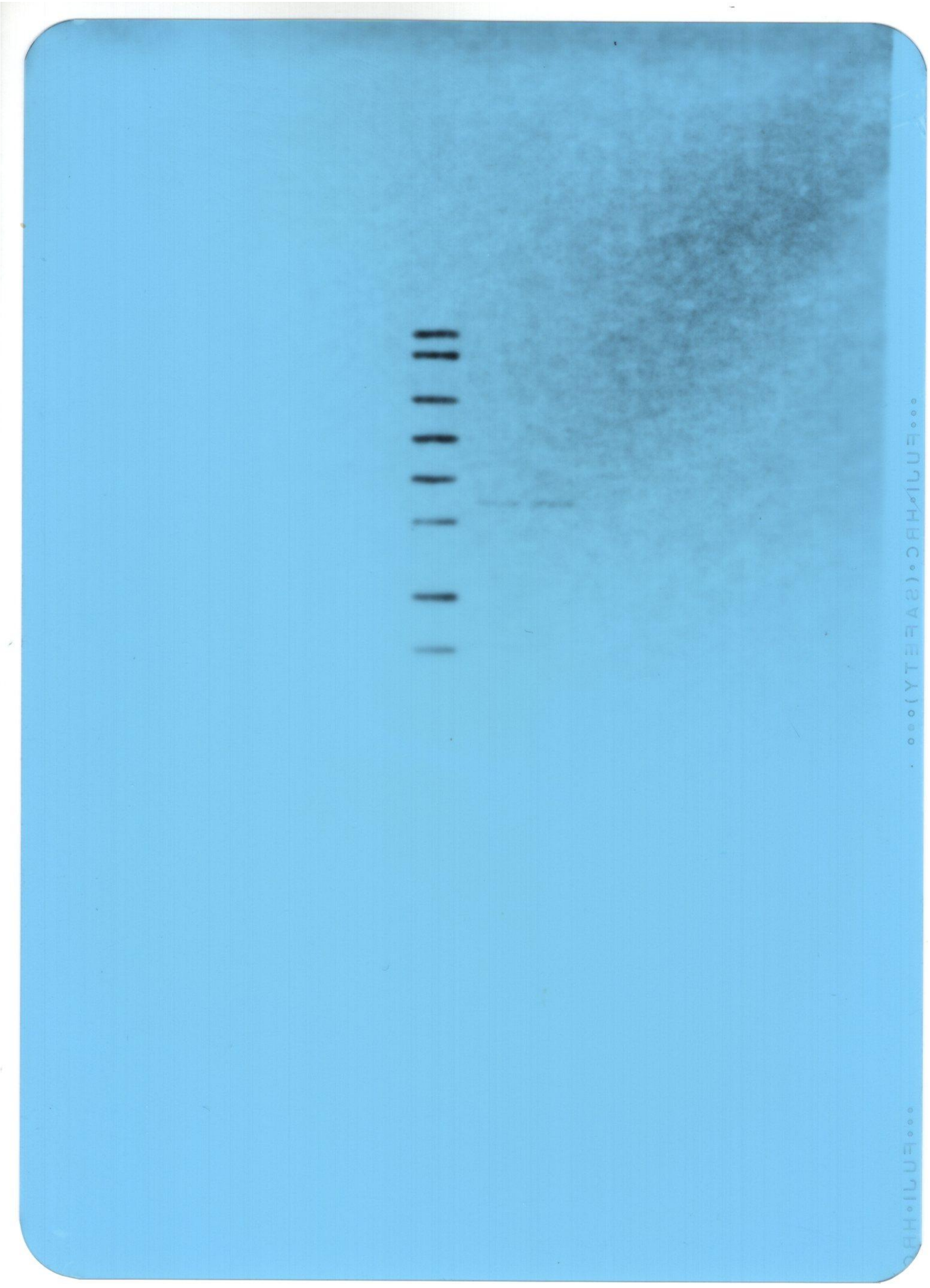

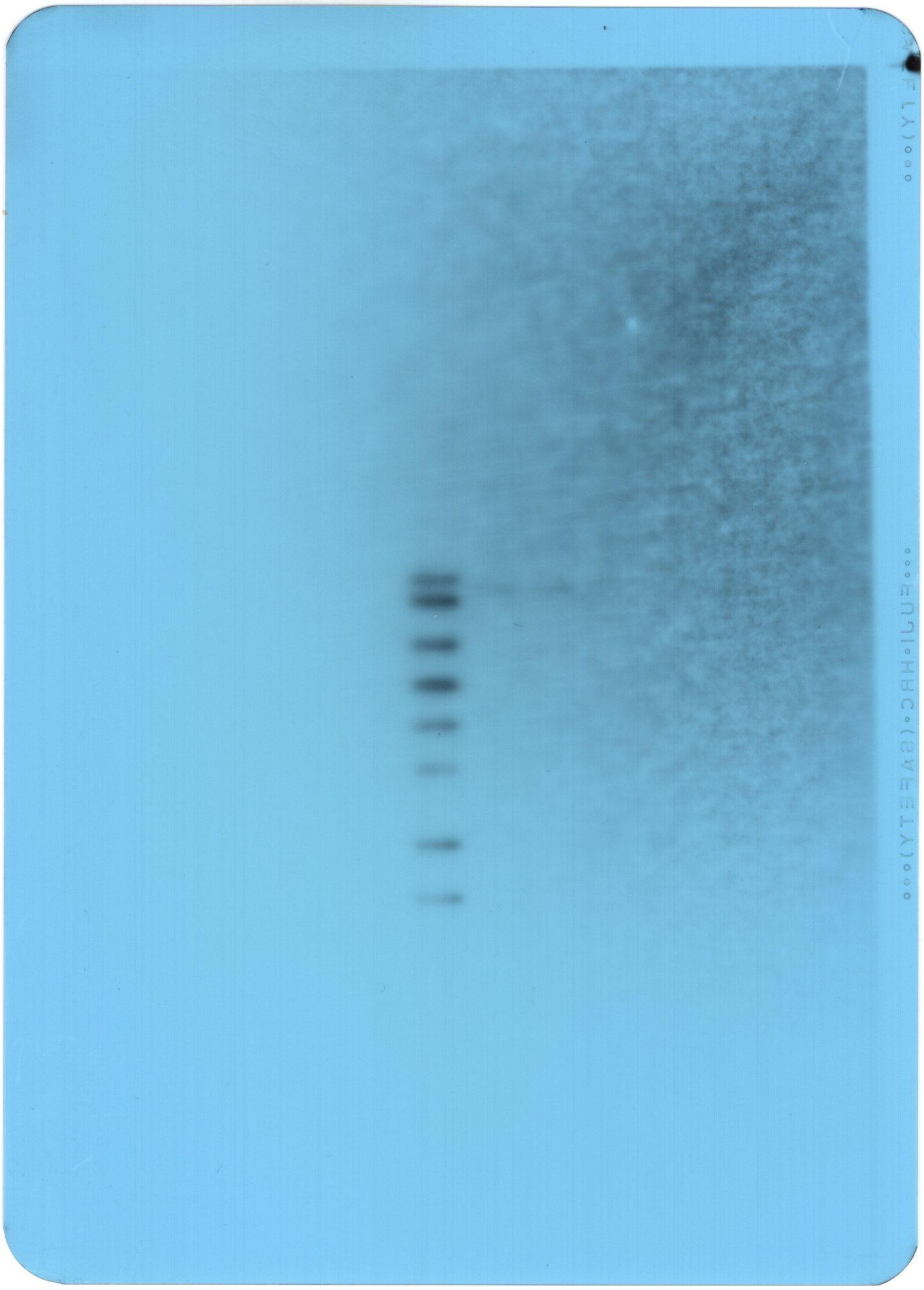

Supplement: Supplementary file 2 — Supplementary Material 2 [file 12885_2024_11855_MOESM2_ESM.pdf]
